# Supplementary material for: Hydrogen-bonded organic framework biomimetic entrapment allowing non-native biocatalytic activity in enzyme
Source: Nat Commun. 2022 Aug 16;13:4816. doi: 10.1038/s41467-022-32454-2 (PMC9381776; doi:10.1038/s41467-022-32454-2)
Supplement: Supplementary file 1 — Supplementary Information [file 41467_2022_32454_MOESM1_ESM.pdf]

Supplementary Information for

Hydrogen-bonded organic framework biomimetic entrapment allowing non-native biocatalytic activity in enzyme

Guosheng Chen[1,†]*, Linjing Tong[1,†], Siming Huang[2], Shuyao Huang[3],Fang Zhu[1], Gangfeng Ouyang[1,3]*

[1]MOE Key Laboratory of Bioinorganic and Synthetic Chemistry, School of Chemistry, Sun Yat-sen University, Guangzhou 510275, China

[2]Guangzhou Municipal and Guangdong Provincial Key Laboratory of Molecular Target & Clinical Pharmacology, the NMPA and State Key Laboratory of Respiratory Disease, School of Pharmaceutical Sciences and the Fifth Affiliated Hospital, Guangzhou Medical University, Guangzhou 511436, China

[3]Instrumental Analysis and Research Center, Sun Yat-sen University, Guangzhou 510275, China

[†]These author contributed equally

*Corresponding authors, E-mail: chengsh39@mail.sysu.edu.cn (G. Chen), cesoygf@mail.sysu.edu.cn (G. Ouyang)

This PDF file includes:

Supplementary Methods
Supplementary Discussion
Supplementary Notes
Supplementary References

1. Supplementary Methods

Reagent and materials.

The enzymes including cytochrome c (Cyt c, from *Equus caballus* heart) and catalase (CAT, from bovine liver) were obtained from Aladdin Chemistry Co., Ltd. (Shanghai, China). Trypsin hydrolase was purchased from Sigma-Aldrich (Shang hai, China).1,3,6,8-Tetrakis (*p*-benzoic acid) pyrene (H4TBAPy, 98%), zinc acetate dihydrate, 2-methyl imidazole (HmIM), imidazole-2-carboxaldehyde, and 4,4'-Diamino-3,3',5,5'-tetramethylbiphenyl (TMB) were purchased from J&K Scientific (Beijing, China). The fluorescence dye of rhodamine b isothiocyanate (RhBTC) was purchased from Macklin Biochemical Technology Co., Ltd. (Shanghai, China). Hydrogen peroxide (H_2O_2) and other organic solvents including dimethyl formamide (DMF), ethanol and acetone were purchased from Guangzhou Chemical Reagent Factory (Guangzhou, China).

Characterization.

Powder X-ray diffraction (PXRD) patterns were collected (0.02°/step, 0.06 seconds/step) on a Bruker D8 Advance diffractometer (Cu Kα) at room temperature. N_2 adsorption isotherms were collected with a JW-DX Surface Area Analyzer at –196 °C. All the samples were pre-treated under 100 °C for 12 h before measurements. The ultraviolet-visible (UV-Vis) absorbance measurements were performed with a 2800S spectrophotometer (SOPTOP, Shanghai), while the UV-Visible diffuse reflectance spectrum was performed with a 3600 spectrophotometer (Shimadzu, Japan). Fourier transform infrared spectrum (FTIR) was carried out on a Bruker EQUINOX 55 spectrometer (32 scans in the 4000-400 cm^{-1} spectral range). Thermogravimetric analyses (TGA) were performed under N_2 atmosphere (20 mL min^{-1}) with temperature increasing at 10 °C min^{-1} using a TA-Q50 system. The samples were dried in vacuo at 100 °C for 12 h before TGA analysis. Solid-state NMR spectra were recorded on Bruker AVANCE III 400MHz spectrometers. The morphology images were analyzed by a SU8010 ultra-high resolution field emission scanning electron microscope (SEM, Hitachi, Japan). Confocal laser scanning microscope (LSM 880 NLO, Carl Zeiss, Göttingen, Germany) was used to determine the distribution of RhB-labelled Cyt c within the nanosystem. Circular dichroism (CD) spectra of Cyt c and

Cyt c@HOF-101 were analyzed by a J1700 CD Spectrometer (JASCO, Japan) in the spectrum region of 190-300 nm. Cryo-EM imaging was performed on an FEI Titan Krios G3i (D3845) TEM operated at 300 kV and equipped with an autoloading mechanism, and the images were taken at a nominal magnification of 350,000 with a pixel size of 0.34 Å by 0.34 Å. The total dose rate was approximately 30 $e^-/Å^2$ for each micrograph. Synchrotron small angle X-ray scattering (SAXS) was collected on the beamline at the Beijing Synchrotron Radiation Facility (BSRF), covering the range of momentum transfer, $q = 4\pi \sin(\theta)/\lambda$, between 0.08 and 3.05 nm^{-1}. EPR experiment was carried out on Bruker EMX plus 10/12 equipped with Oxford ESR910 Liquid Helium cryostat. The test temperature was set at 5 K.

Preparation of standard HOF-101.

The standard HOF-101 was prepared according to the reported method[1]. 150 mg H4TBAPy (0.225 mmol) was dissolved in 22.5 mL of DMF to which 90 mL MeOH was added, and then stirred for 1 min. The mixture was stood at room temperature for 12 h to afford yellow crystals of HOF-101. Finally, the products were collected by centrifugation, washed with ethanol three times and dried under vacuum at room temperature. This sample was used as a standard for a comparison with the Cyt c@HOF-101.

Surface-adsorption experiment. To demonstrate that the Cyt c was indeed encapsulated into, rather than surface-adsorbed onto HOF-101, we carried out this surface-adsorption experiment. We first examined the surface adsorption capacity of HOF-101 towards Cyt c. The relative narrow mesopore of HOF-101 (ca. 2.0 nm) is not insufficient to accommodate the bulky Cyt c (the molecular dimension of Cyt c is ca. 3.2 nm × 2.7 nm × 3.8 nm, Supplementary Fig. 3a). Given this, 5 mg Cyt c was dispersed in 10 mL as-synthesized standard HOF-101 solution (1 mg/mL). After 15 min stirring (We chose 15 min herein, because the time for the de novo assembly of Cyt c@HOF-101 is 15 min), the Cyt c-adsorbed HOF-101 was collected, and the surface-adsorbed enzymes by HOF-101 were evaluated based on the concentration changes in the supernatants before and after adsorption. The UV-Vis spectra of the collected supernatants showed that almost no Cyt c

was adsorbed by the HOF-101 (Supplementary Fig. 3b). In addition, the apparent color of the Cyt c-adsorbed HOF-101 was consistent with that of the pure HOF-101. But the color of the Cyt c@HOF-101 turned into brown, because of the incorporation of Cyt c (Supplementary Fig. 3c). Furthermore, the FT-IR data also implied that no characteristic peaks of Cyt c were recorded in HOF-101 after the adsorption process (Supplementary Fig. 3d). On the contrary, obvious amide I (1700-1610 cm^{-1}) and amide II (1595-1480 cm^{-1}) bands of Cyt c were appeared in Cyt c@HOF-101 using the biomineralization method.

Fluorescence labeling

Fluorescence labeling of enzymes was based on the conjugation between the amino of lysine residue of enzymes and the thiocarbamide of rhodamine b isothiocyanate (RhBTC, a red fluorescence dye). In brief, 20 mg Cyt c was dispersed into 10 mL carbonate buffer solution (pH=9.0, 0.5 M), followed by adding 1 mg RhBTC. The mixed solution was then stirred for 12 h in the dark. Finally, the RhB-labelled Cyt c was obtained through ultrafiltration by a centrifugal filter device (molecular weight cut-off MWCO= 8 kDa) 3 times to remove excess reagents and salts.

Preparation of Cyt c@NU-1000 nanosystem

The Cyt c@NU-1000 nanosystem was synthesized through a post-infiltration method[2,3]. NU-1000 was firstly prepared and activated according to the reported method[4]. The small size of Cyt c molecule (ca. 13 kDa, 3.2 nm × 2.7 nm × 3.8 nm) allowed it to infiltrate into the mesopores (ca. 3.2 nm) of NU-1000 in the specific orientation. For Cyt c infiltration, 1.5 mg of Cyt c and 7.5 mg of NU-1000 were dispersed in 7.5 mL of 0.90 M Tris buffer (pH=7.5). The mixed system was left on an incubator shaker at 350 rpm for 24 h at room temperature. The precipitate was collected by centrifugation at 8000 rpm, and then washed, sonicated, and centrifuged three times to remove loosely adsorbed Cyt c. Finally, the products were dried by lyophilization.

Preparation of Cyt c@ZIF-8 nanosystem

The Cyt c@ZIF-8 nanosystem was synthesized through a de novo encapsulation strategy[5].

2 mg Cyt c was dispersed into 1 mL deionized water. The enzymes solution was then stirred for 10 s, followed by adding 2 mL zinc acetate solution (0.1 M) and 2 mL HmIM solution (1.2 M). The mixture was aged for 4 h at room temperature. The formed Cyt c@ZIF-8 were collected by centrifugation at 8000 rpm, and then washed, sonicated, and centrifuged three times to remove loosely adsorbed Cyt c. Finally, the products were dried by lyophilization.

Preparation of Cyt c@ZIF-90 nanosystem

The Cyt c@ZIF-90 nanosystem was also synthesized through a *de novo* encapsulation strategy[6]. 48 mg imidazole-2-carboxaldehyde was heated to dissolve in 2.5 mL deionized water. Then 1.0 mg Cyt c and 37.1 mg zinc nitrate hexahydrate were added. The resultant mixture was stirred for 10 min at room temperature. Finally, the precipitate was collected by centrifugation at 8000 rpm, and then washed, sonicated, and centrifuged three times to remove loosely adsorbed Cyt c. Finally, the products were dried by lyophilization.

Measurement of the Cyt c content in the nanosystem.

Bradford protein assays

The Cyt c content in the nanosystem (Supplementary Table 1) was measured by examining the concentration differences of enzymes in the supernatants before and after assembly via Bradford protein assays[7]. Typically, 20 μL Cyt c sample was added to a 96-well plate, followed by introducing 200 μL Coomassie Brilliant Blue G-250 reagent. After 5 min incubation, the solution was collected and detected by UV-Vis spectrophotometer. The concentration of Cyt c is proportional to the Abs at 595 nm (Supplementary Fig. 2a).

Inductively coupled plasma-mass spectrometry

Cyt c is a heme protein (ca. 13 KD) that contains one Fe atom per protein. Inductively coupled plasma-mass spectrometry (ICP-MS) was performed to further evaluate the enzyme loading of Cyt c@HOF-101 by examining the content of Fe in the biocomposites. Briefly, 0.15 mg Cyt c@HOF-101 was digested by 100 μL concentrated nitric acid for 2 h at 120 °C. The digested supernatant was collected, and the concentration of Fe was

quantified by the standard calibration curve (Supplementary Fig. 2b). The Cyt c loading of Cytc@HOF-101 could be calculated based on the Fe concentration.

Examination of CAT activity of Cyt c nanosystem.

CAT catalyzes the decomposition of H_2O_2 into water and oxygen[8] (Equation 1):

$$2H_2O_2 \xrightarrow{CAT} O_2 + 2H_2O \qquad \text{(Equation 1)}$$

The CAT-like activity of the Cyt c nanosystem was measured by the time-dependent production of oxygen, and the dosages of Cyt c in each trial including free Cyt c, Cyt c@ZIF-8, Cyt c@ZIF-90, Cyt c@NU-1000 and Cyt c@HOF-101 groups were set at 0.1 mg/mL, and the dosages used of different Cyt c nanosystems were according to the calculated enzymes loading by the Bradford assays. Briefly, the Cyt c nanosystem was dispersed into 4.5 mL Tris buffer (pH 7.5, 50 mM), and the mixed solution was then degassed by bubbling with nitrogen for 20 min. Finally, 500 μL of 100 mM H_2O_2 was rapidly added to initiate the reaction. The catalytic reaction was carried out in an enclosed environment, wherein the probe of the dissolved oxygen analyzer (JBP-607A, Shanghai, China) was immersed into the solution to real-time record the generation of oxygen.

Examination of peroxidase activity of Cyt c@HOF-101.

The peroxidase activity of Cyt c was evaluated using TMB as the substrates (Equation 2)

$$H_2O_2 + TMB \xrightarrow{Cytc} H_2O + oxTMB \qquad \text{(Equation 2)}$$

The concentrations of Cyt c in each trial including free Cyt c and Cyt c@HOF-101 groups were set at 6.67 μg/mL. In a typical test, the free Cyt c or Cyt c@HOF-101 were dispersed into 0.1 mL Tris buffer (pH 7.5, 50 mM), followed by adding 0.2 mL prepared TMB solution (0.3 mg/mL) as the hydrogen donor. Immediately, 0.2 mL of 10 mM H_2O_2 was added to activate the catalytic reaction. The generated oxTMB could be traced at 652 nm by a UV-Vis spectrophotometer using a time-scanning mode.

Measurement of the catalytic kinetic parameters

Enzymatic kinetics was evaluated according to the Michaelis-Menten equation[9] (Equation

3):

$$V_0 = \frac{V_{max}[S]}{K_m+[S]} \qquad \text{(Equation 3)}$$

Here, V_0 is the initial catalytic rate; V_{max} is the maximum rate conversion, which is obtained when the catalytic sites on the enzyme are saturated with substrate. [S] is the initial substrate concentration, and K_m is the Michaelis-Menten constant. The initial catalytic rates V_0 was determined by making the slope of the kinetic curve in the initial phase, and the initial substrate concentration [S] was determined at t = 0 s. The kinetic parameters K_m and V_{max} were fitted using a Michaelis–Menten equation based on the calculated V_0 and [S]. The analysis was performed with the Software of GraphPad prism version 5.0.0 (GraphPad Software, Inc., San Diego, CA).

2. Supplementary Discussion

The simulation setup of Cyt c-HOF-101 system for molecular dynamics simulations. Our previous work has demonstrated that proteins can trigger the HOF-101 nucleation around theirs surface[10] (Supplementary Fig. 1), and this results in a defective cavity for the accommodation of proteins. Cyt c has a spherical structure, with a maximum diameter of 3.84 nm (Supplementary Fig. 20a). Hence, a defective cavity of at least 3.84 nm was subsequently set in HOF-101 for accommodating the Cyt c. We note that the central axis between HOF-101 monomers shows a planar structure, and the hydrogen bond is formed between the carboxylate of adjacent monomers, and the distance between the hydrogen bond is 0.33 nm. In addition, the distance between the C atoms on the carboxylate of the two monomer side chains is 2.16 nm. Considering the maximum diameter of sphere-like Cyt c is 3.84 nm, we set up a tetramer with defective structure as shown in Supplementary Fig. 20b. This tetramer has a ca. 4.5 nm sphere-like cavity, which enables the accommodation of Cyt c.

In the docking process, 8-layer tetramer of defective HOFs-101 was used as the receptor and Cyt c molecule was used as the ligand. A total of 50 kinds of Cyt c@HOF-101 phases were obtained by the docking process (Supplementary Table 4). The docking results showed that Cyt c was extremely concentrated in the defective HOF-101, and the docking phase was also highly concentrated (Supplementary Fig. 21). It was observed that 80% of the energy was concentrated in the range of ca. -160.0 kJ/mol to ca. -157.0 kJ/mol (Supplementary Table 4). The Cyt c@HOF-101 system with the largest docking cluster and the lowest energy was selected for the following dynamic analysis.

3. Supplementary Notes

Supplementary Tables

Supplementary Table 1. The encapsulation efficiencies of Cyt c in different materials.

Nanosystem	Cyt c content[a]
Cyt c@HOF-101	39 wt%
Cyt c@NU-1000	11 wt%
Cyt c@ZIF-8	5 wt%
Cyt c@ZIF-90	3 wt%

a.The Cyt c content was evaluated by examining the concentration differences of the enzyme in the supernatants before and after assembly by the standard Bradford assays.

Supplementary Table 2. Step-by-step summarization of the experimental details for CAT-like activity and peroxidase activity tests

Activity test	Step 1	Step 2	Step 3
**CAT-like activity**	Free Cyt c, Cyt c@ZIF-8, Cyt c@ZIF-90, Cyt c@NU-1000 or Cyt c@HOF-101 was dispersed into 4.5 mL Tris buffer (pH 7.5, 50 mM) in a plastic tube, respectively. The final Cyt c concentration in each trial was kept at 0.1 mg/mL.	The mixed solution was then degassed by bubbling with high purity nitrogen for 20 min	500 μL of 100 mM H_2O_2 was rapidly added to initiate the reaction under static state in an enclosed environment. The produced O_2 was real-time measured by dissolved oxygen analyzer (JBP-607A, Shanghai, China)
**peroxidase activity**	The free Cyt c or Cyt c@HOF-101 was dispersed into 0.1 mL Tris buffer (pH 7.5, 50 mM) in an ultraviolet cuvette. The final Cyt c concentration in each trial was kept at 6.67 μg/mL.	0.2 mL prepared TMB solution (0.3 mg/mL) was added as the hydrogen donor.	Immediately, 0.2 mL of 10 mM H_2O_2 was added to activate the catalytic reaction under static state. The generated oxTMB could be traced at 652 nm by a UV-Vis spectrophotometer using a time-scanning mode.

Supplementary Table 3. Step-by-step summarization of the experimental details for the stability tests

Stability test	Step 1	Step 2	Step 3
**Stability against pH**	Free CAT (1 μg/mL) or biocomposites (0.1 mg/mL) were exposed in 4.5 mL deionized water with different pH (pH=2, 4, 6, 8) at room temperature for 30 min, respectively.	After exposure, the solution was degassed by bubbling with high purity nitrogen for 20 min.	500 μL of 100 mM H_2O_2 was rapidly added to initiate the reaction under static state in an enclosed environment. The O_2 production rate, which was evaluated by the slope of the kinetic curve in the initial phase from 0 to 40 s, was used for the evaluation of activity change.
**Stability against heating treatment**	Free CAT (1 μg/mL) or biocomposites (0.1 mg/mL) were dispersed in 4.5 mL pH=7 deionized water in a plastic tube, and then exposed at 60 °C, 80 °C and 100 °C for 30 min, respectively.	After exposure, the solution was degassed by bubbling with high purity nitrogen for 20 min.	500 μL of 100 mM H_2O_2 was rapidly added to initiate the reaction under static state in an enclosed environment. The O_2 production rate, which was evaluated by the slope of the kinetic curve in the initial phase from 0 to 40 s, was used for the evaluation of activity change.
**Stability against denaturing regents and organic solvents**	Free CAT (1 μg/mL) or biocomposites (0.1 mg/mL) were exposed in 4.5 mL different solutions in a plastic tube at room temperature for 30 min, respectively. Urea: 6 mol/L; Trypsin: 5 mg/mL; All of the metal ions: 10 mmol/L; Organic solvents: 80% (v/v).	After exposure, the solution was degassed by bubbling with high purity nitrogen for 20 min.	500 μL of 100 mM H_2O_2 was rapidly added to initiate the reaction under static state in an enclosed environment. The O_2 production rate, which was evaluated by the slope of the kinetic curve in the initial phase from 0 to 40 s, was used for the evaluation of activity change.
**Storage Stability**	Free CAT (1 μg/mL) or biocomposites (0.1 mg/mL) were dispersed in 4.5 mL pH=7 deionized water in a plastic tube, and then stood at 35 °C for 1 d, 2 d, 3d ,4d and 5d, respectively.	After placing for different times, the solution was degassed by bubbling with high purity nitrogen for 20 min.	500 μL of 100 mM H_2O_2 was rapidly added to initiate the reaction under static state in an enclosed environment. The O_2 production rate, which was evaluated by the slope of the kinetic curve in the initial phase from 0 to 40 s, was used for the evaluation of activity change.

Supplementary Table 4. The conformations of the simulated Cyt c@HOF-101

The docking energy of 50 conformations of Cyt c@HOF-101 system			
**Docking pose**	**Docking energy**	**Docking pose**	**Docking energy**
Conformation1	-157.5 kJ/mol	Conformation26	-157.5 kJ/mol
Conformation2	-160.4 kJ/mol	Conformation27	-161.0 kJ/mol
Conformation3	-157.5 kJ/mol	Conformation28	-157.5 kJ/mol
Conformation4	-157,9 kJ/mol	Conformation29	-157.6 kJ/mol
Conformation5	-157.7 kJ/mol	Conformation30	-157.7 kJ/mol
Conformation6	-159.8 kJ/mol	Conformation31	-160.9 kJ/mol
Conformation7	-159.8 kJ/mol	Conformation32	-157.7 kJ/mol
Conformation8	-157.6 kJ/mol	Conformation33	-157.7 kJ/mol
Conformation9	-157.6 kJ/mol	Conformation34	-160.4 kJ/mol
Conformation10	-157.5 kJ/mol	Conformation35	-157.7 kJ/mol
Conformation11	-157.5 kJ/mol	Conformation36	-160.5 kJ/mol
Conformation12	-157.8 kJ/mol	Conformation37	-157.5 kJ/mol
Conformation13	-157.9 kJ/mol	Conformation38	-157.6 kJ/mol
Conformation14	-157.5 kJ/mol	Conformation39	-157.5 kJ/mol
Conformation15	-157.6 kJ/mol	Conformation40	-157.5 kJ/mol
Conformation16	-157.7 kJ/mol	Conformation41	-157.7 kJ/mol
Conformation17	-157.6 kJ/mol	Conformation42	-157.5 kJ/mol
Conformation18	-157.6 kJ/mol	Conformation43	-157.7 kJ/mol
Conformation19	-157.6 kJ/mol	Conformation44	-157.6 kJ/mol
Conformation20	-157.6 kJ/mol	Conformation45	-160.5 kJ/mol
Conformation21	-157.5 kJ/mol	Conformation46	-160.5 kJ/mol
Conformation22	-157.6 kJ/mol	Conformation47	-157.5 kJ/mol
Conformation23	-157.8 kJ/mol	Conformation48	-157.7 kJ/mol
Conformation24	-157.6 kJ/mol	Conformation49	-157.6 kJ/mol
Conformation25	-160.9 kJ/mol	Conformation50	-157.6 kJ/mol

It was observed that 80% of the energy was concentrated in the range of ca. 160.0 kJ/mol to ca. 157.0 kJ/mol, and the docking phase was highly concentrated (Supplementary Fig. 21). The Cyt c@HOF-101 system with the largest docking cluster and the lowest energy was selected for the following kinetic analysis.

Supplementary Figures

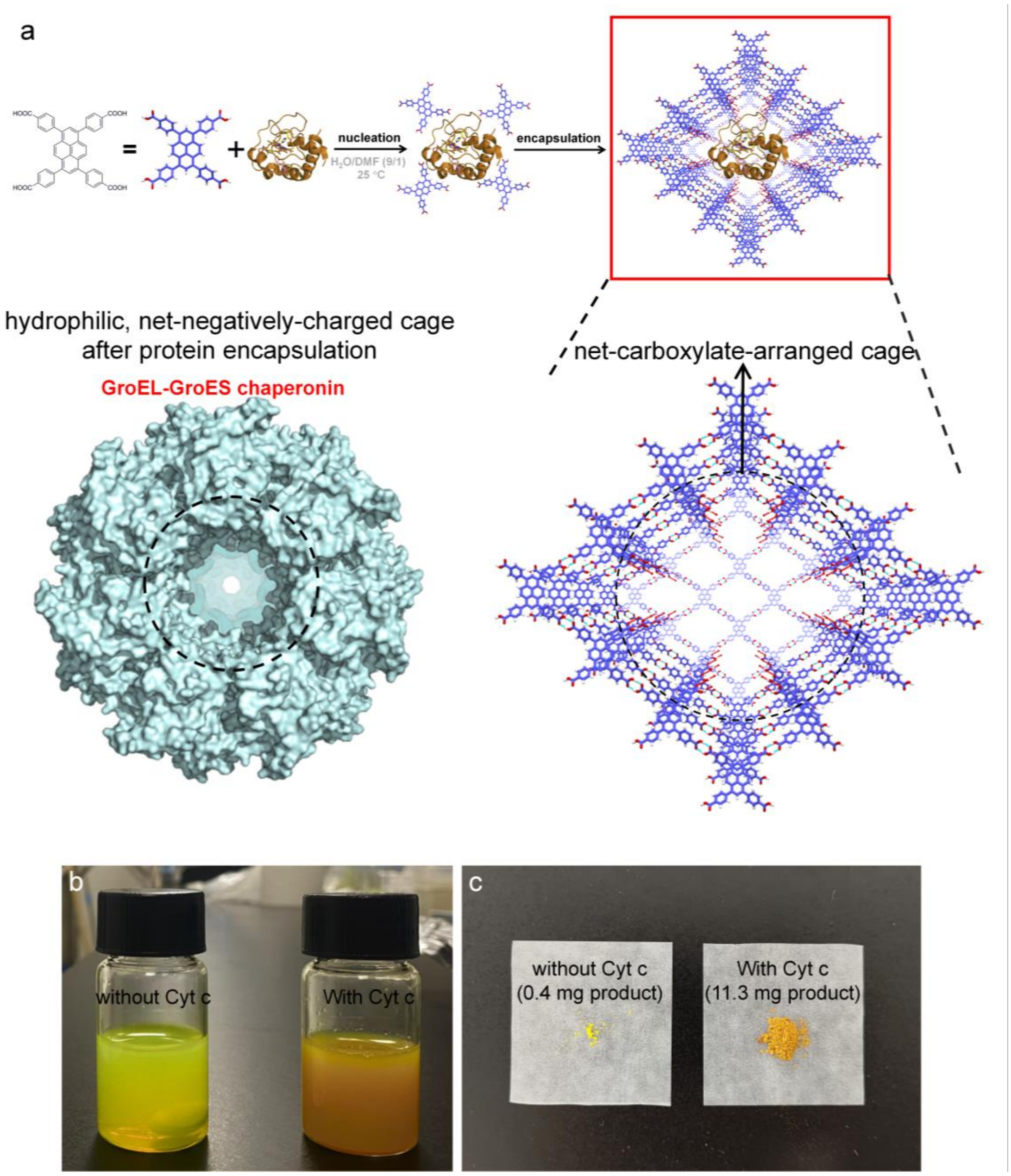

Supplementary Fig. 1. Synthesis of Cyt c@HOF-101 nanosystem. (a) The principle of the preparation of Cyt c@HOF-101 naonosystem. Cyt c was able to trigger the HOF-101 nucleation around its surface, and then a net-carboxylate-arranged defective cage was formed for the accommodation of Cyt c. Such net-carboxylate-arranged defective cage resulted in a highly hydrophilic, net-negatively-charged microenvironment, as like the chaperone cage (PDB: 1pf9). (b) Digital photographs recorded the assembly process with Cyt c and without Cyt c. (c) The yields of the products. It was clearly observed that Cyt c accelerated the formation of HOF-101, whereas almost no product was formed without the addition of Cyt c. The resultant HOF-101 biocomposite turned into brownness because of the encapsulation of Cyt c.

a

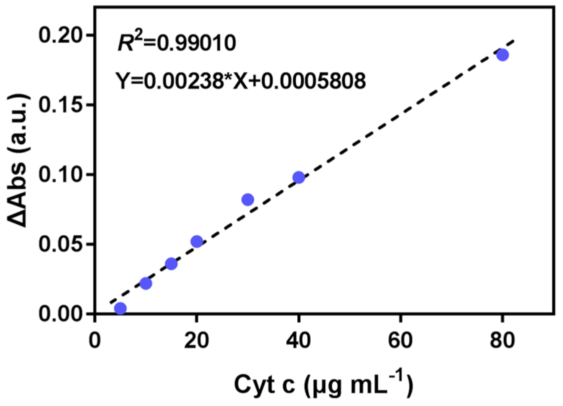

b

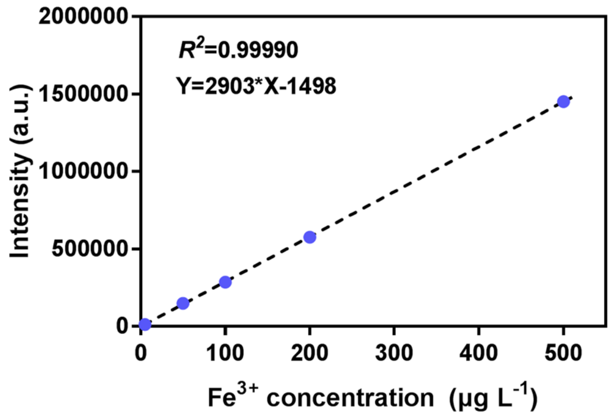

c

Sample (Cyt c@HOF-101)	NO.1	NO.2	NO.3	NO.4	NO.5	Average
Fe（wt%)	0.153	0.151	0.155	0.178	0.144	0.156
Cyt c (wt%)	35.56	35.00	35.95	41.29	33.36	36.23

Supplementary Fig. 2. Cyt c quantification. (a) The standard curve for Cyt c quantification using Bradford proteins assays; (b) The standard curve of Fe ion for Cyt c quantification using ICP-MS, and (c) the calculated Cyt c loadings in five batches of Cyt c@HOF-101.

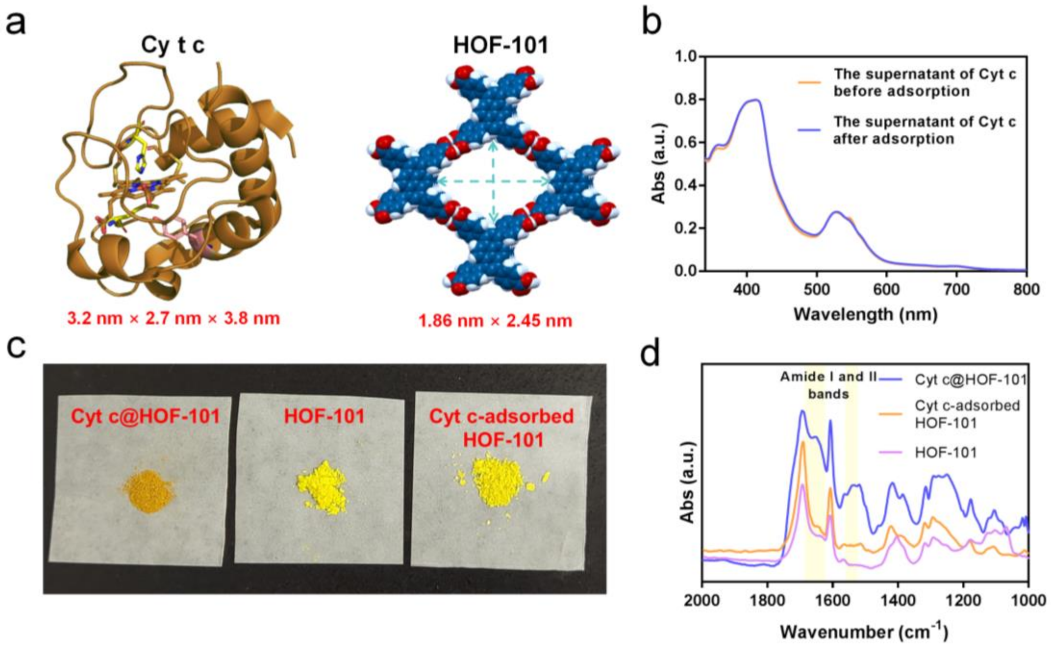

Supplementary Fig. 3. The surface-adsorption experiment. (a) The molecular dimensions of Cyt c based on PyMOL Molecular Graphics System (Version 2.4.0), and the mesopore structure of HOF-101; (b) The UV-Vis spectra of the collected supernatants before and after adsorption experiment; The apparent color (c) and FT-IR spectra (d) of Cyt c@HOF-101, HOF-101 and Cyt c-adsorbed HOF-101, respectively.

The relative narrow mesopore in HOF-101 (ca. 2.0 nm) is not insufficient to accommodate bulky Cyt c (the molecular dimension of Cyt c is ca. 3.2 nm × 2.7 nm × 3.8 nm, Supplementary Fig. 3a). In the surface-adsorption experiment, the UV-Vis spectra of the collected supernatants showed that almost no Cyt c was adsorbed by the HOF-101 (Supplementary Fig. 3b). In addition, the apparent color of the Cyt c-adsorbed HOF-101 was consistent with the pure HOF-101, while the color of the Cyt c@HOF-101 changed from yellow to brown, because of the incorporation of Cyt c (Supplementary Fig. 3c). Furthermore, the FT-IR data also implied that no characteristic peaks of Cyt c were recorded in HOF-101 after the adsorption process (Supplementary Fig. 3d). On the contrary, obvious amide I (1700-1610 cm^{-1}) and amide II (1595-1480 cm^{-1}) bands of Cyt c were appeared in Cyt c@HOF-101. The aforementioned together demonstrated that the surface adsorption of Cyt c by HOF-101 was very limited.

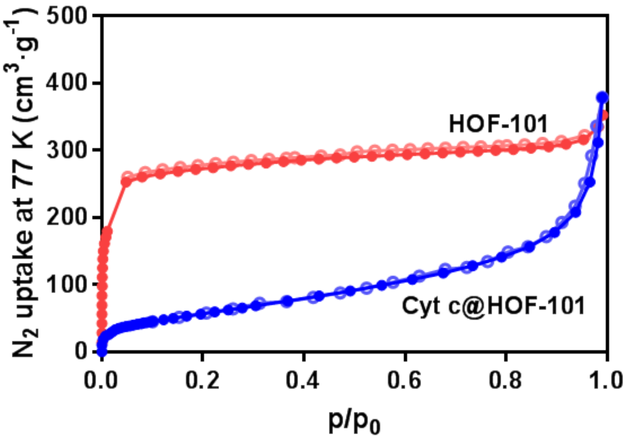

Supplementary Fig. 4. The nitrogen adsorption/desorption isotherms of HOF-101 and Cyt c@HOF-101. The N_2 adsorption amount of Cyt c@HOF-101 was significantly lower than that of the HOF-101, suggesting that the plentiful pores of HOF-101 were occupied by the encapsulated Cyt c.

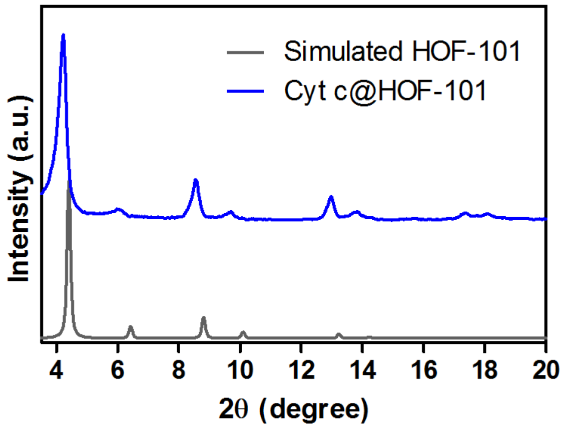

Supplementary Fig. 5. The crystallinity of Cyt c@HOF-101. The PXRD patterns of simulated HOF-101 and Cyt c@HOF-101. The Cyt c@HOF-101 well inherited the Bragg diffraction peaks of HOF-101, suggesting the high crystallinity of Cyt c@HOF-101.

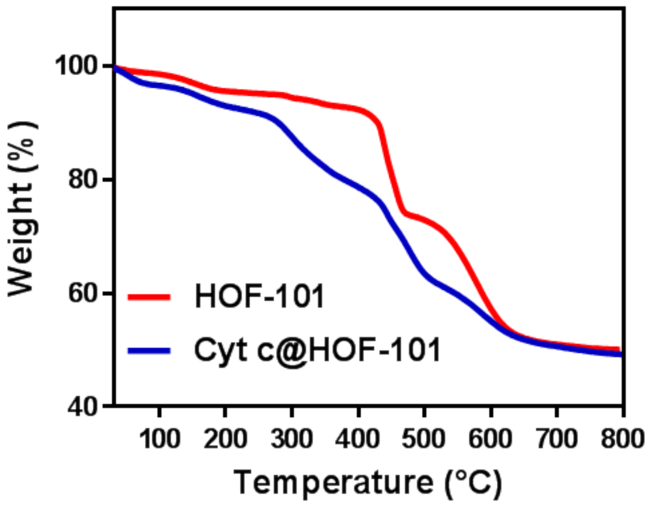

Supplementary Fig. 6. The TGA analysis. The TGA of HOF-101 and Cyt c@HOF-101, respectively. The extra weight loss of Cyt c@HOF-101 between ca. 250°C to 450°C was caused by the pyrolysis of the incorporated Cyt c.

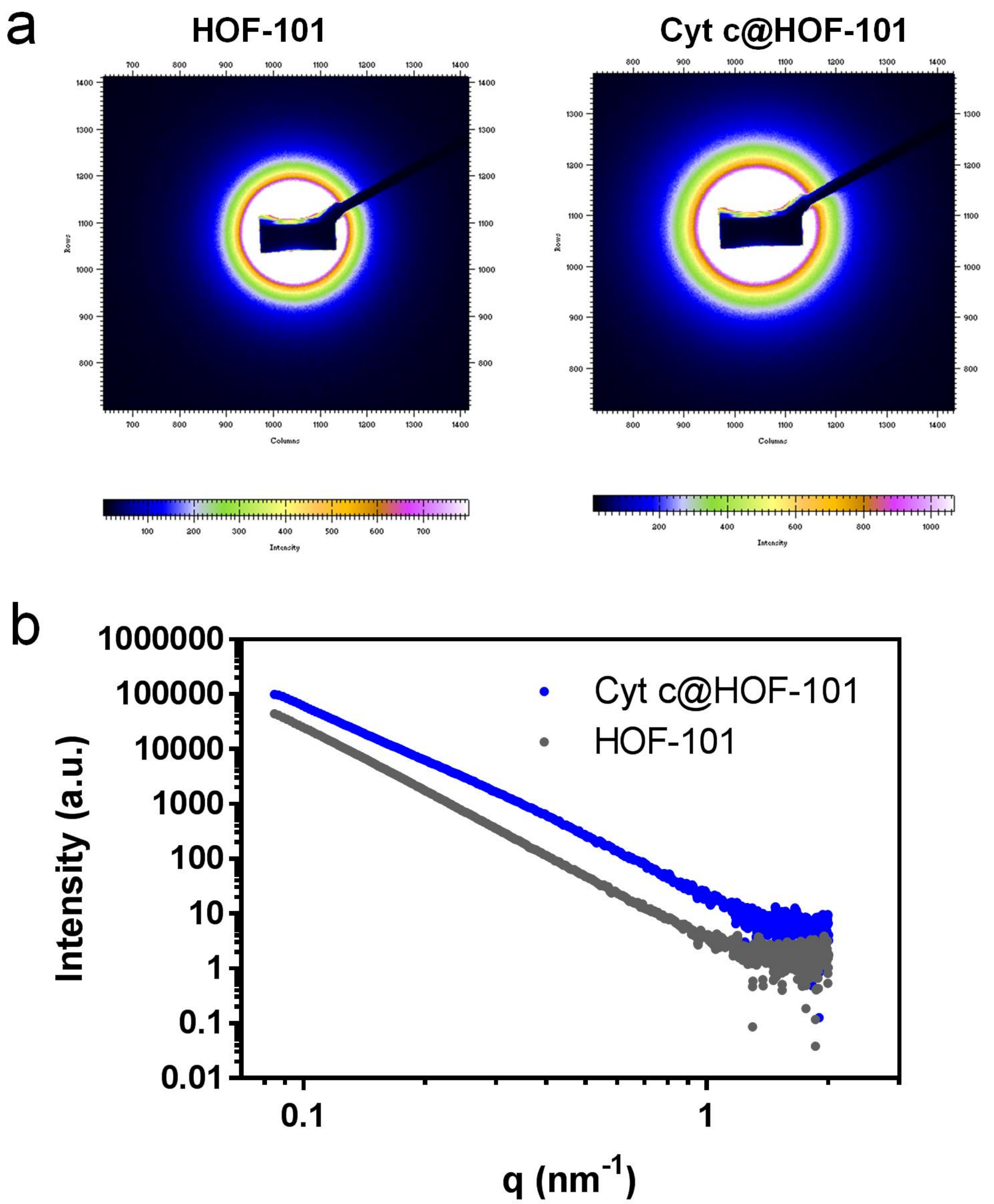

Supplementary Fig. 7. The SAXS analysis. The 2D (a) and 1D SAXS patterns (b) of HOF-101 and Cyt c@HOF-101, respectively. The higher scattering intensity of Cyt c@HOF-101 in the low q region (ranged from 0.1 to 1 nm^{-1}) suggested the high structural inhomogeneity. This was caused by the internalization of Cyt c in Cyt c@HOF-101.

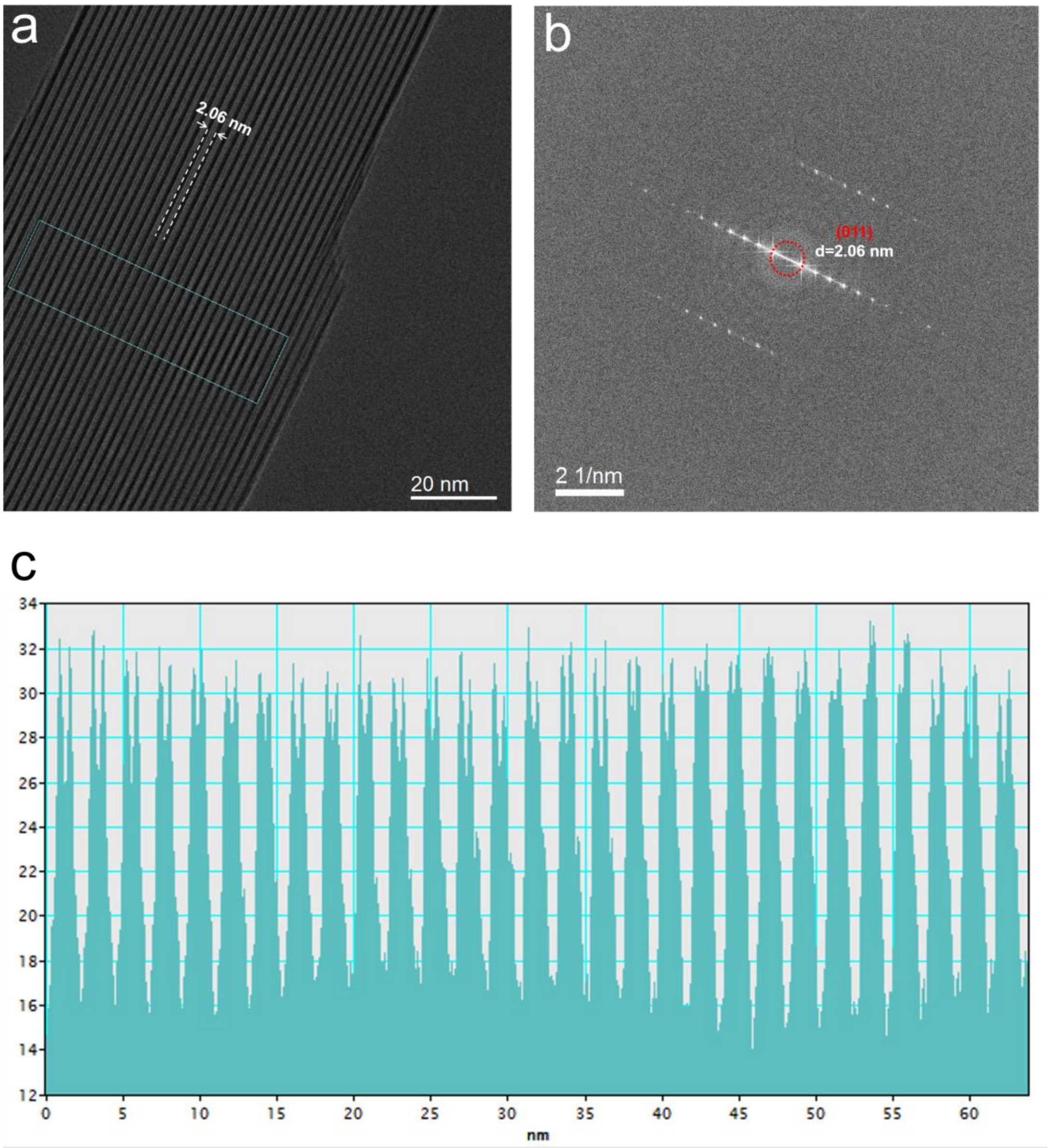

Supplementary Fig. 8. The typical Cryo-EM images of randomly selected Cyt c@HOF-101. (a) The structural profile of Cyt c@HOF-101 by cryo-EM viewing from the $[01\bar{1}]$ projection, and (b) the corresponding FFT pattern. (c) The average intensity profile from the selected area (green) in (a). It showed that the prepared Cyt c@HOF-101 had long-range ordered pores across the whole crystallite.

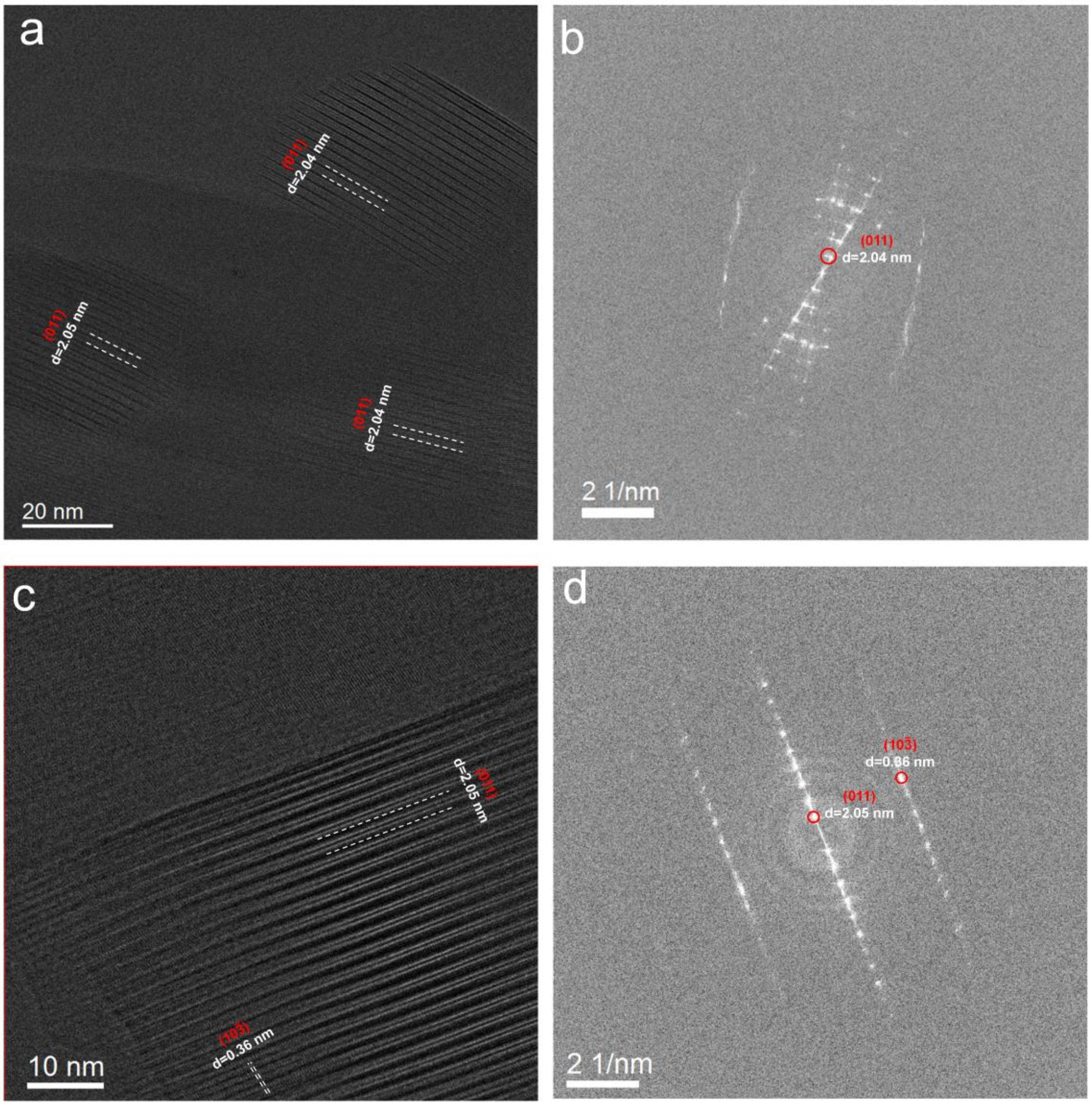

Supplementary Fig. 9. The typical cryo-EM images of randomly selected Cyt c@HOF-101. (a) The structural profile by cryo-EM showed the lattice plane of (011) in different Cyt c@HOF-101 crystals, and (b) the corresponding FFT pattern. (c) The structural profile by cryo-EM showed the lattice planes of (011) and (10$\bar{3}$) in a Cyt c@HOF-101 crystal, and (d) the corresponding FFT pattern.

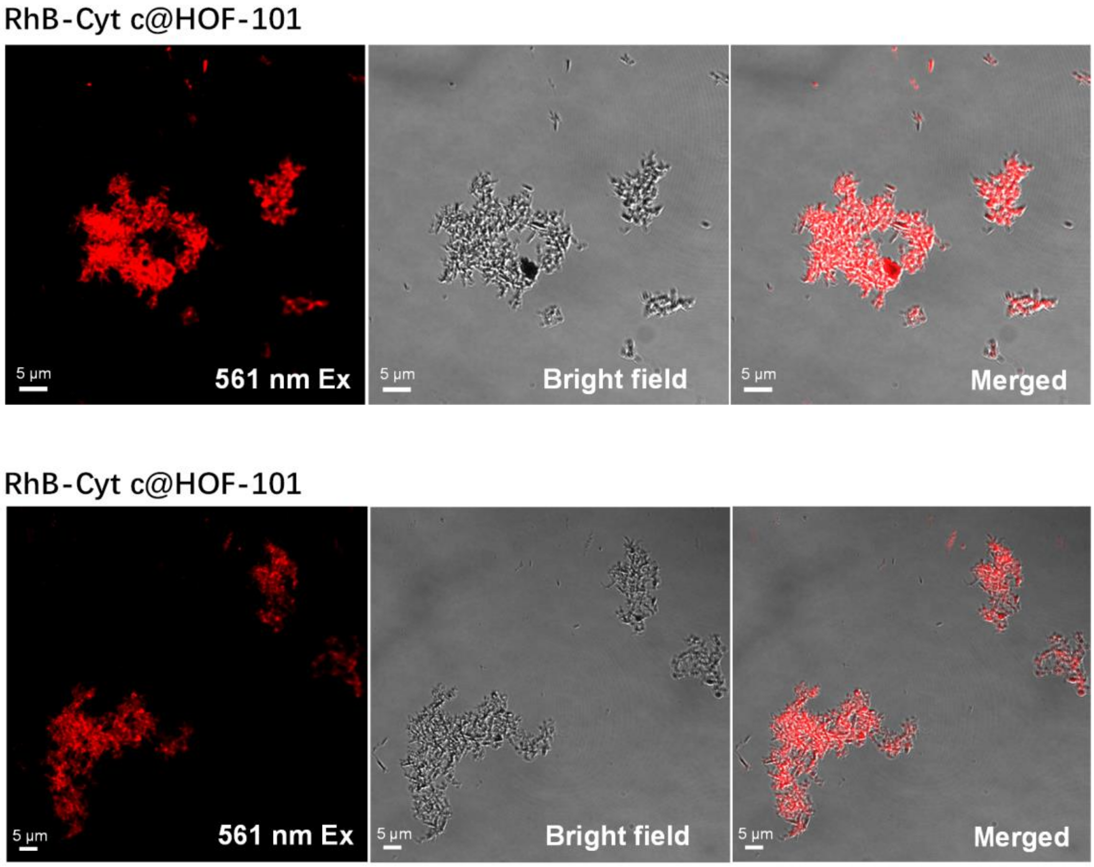

Supplementary Fig. 10. The spatial distribution of Cyt c within HOF-101. The CLSM images of Cyt c@HOF-101, wherein the Cyt c was labelled with RhB (Rhodamine B). The below images were also presented in Fig. 1e in main text. The red fluorescence completely overlaid with the whole frameworks, suggesting the uniformly spatial distribution of Cyt c within HOF-101.

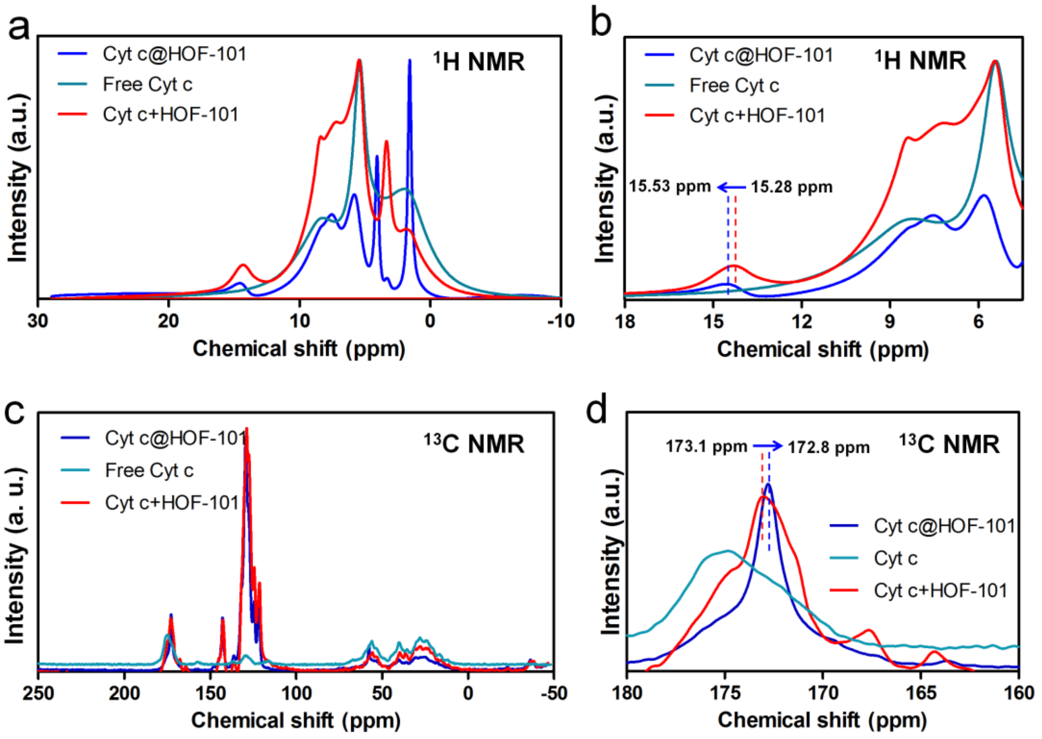

Supplementary Fig. 11. The solid-state NMR (ssNMR) spectra. (a) The full ^{1}H ssNMR spectra of Cyt c@HOF-101, Cyt c, and the physical mixture of Cyt c and HOF-101 (Cyt c+HOF-101). (b) The amplified ^{1}H ssNMR spectra in (a). (c) The full ^{13}C ssNMR spectra of Cyt c@HOF-101, Cyt c, and the physical mixture of Cyt c and HOF-101 (Cyt c+HOF-101). (d) The amplified ^{13}C ssNMR spectra in (c). The ssNMR spectrum of Cyt c@HOF-101 was different from that of the Cyt c+HOF-101. Specially, the chemical shifts of ^{1}H ssNMR ranged from 14 to 15 ppm and of ^{13}C ssNMR ranged from 170 to 175 ppm, which were assigned to the –COOH group of HOF-101, were observed to be shifted in Cyt c@HOF-101. It suggested that the interfacial interaction between HOF-101 and Cyt c was formed in Cyt c@HOF-101.

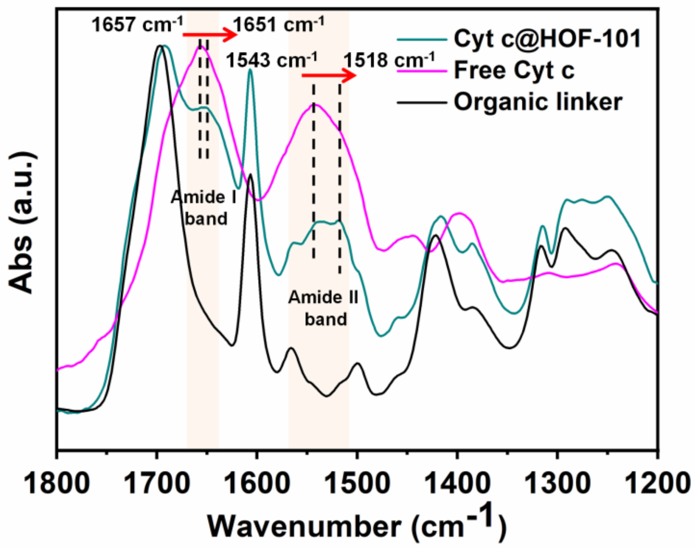

Supplementary Fig. 12. Insight into the interfacial interaction by FT-IR. The FI-IR spectra of Cyt c@HOF-101, Cyt c, and organic linker of HOF-101. The red-shifts of amide I and II bands of Cyt c in Cyt c@HOF-101 further elucidated the interfacial interaction between Cyt c and HOF-101.

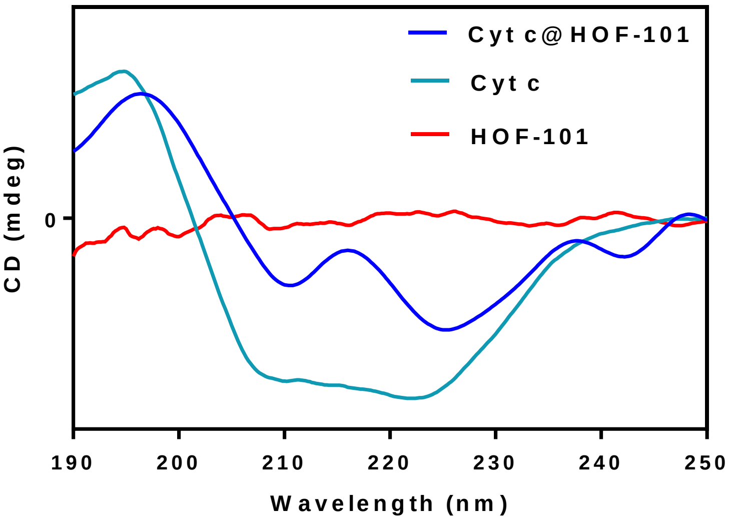

Supplementary Fig. 13. The secondary structure of Cyt c. The CD spectra of Cyt c@HOF-101, Cyt c, and HOF-101, respectively. The free Cyt c showed typical features of α-helix structure with two distinct negative bands at 208 and 222 nm. We observed that the Cyt c@HOF-101 well retained these characteristic CD bands, and the relatively low CD signal was due to the light scattering of nanoparticle.[11] The CD data verified that the secondary structure of Cyt c in Cyt c@HOF-101 was well maintained.

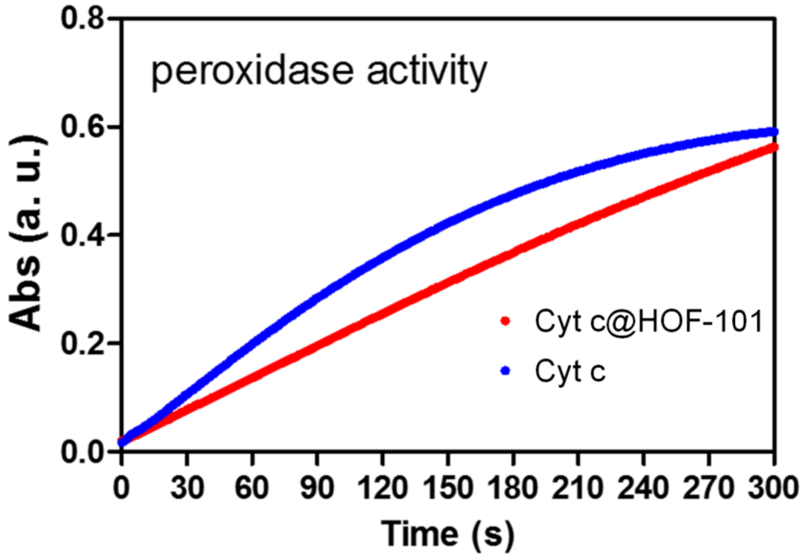

Supplementary Fig. 14. The peroxidase activity of Cyt c@HOF-101. The peroxidase activity of Cyt c@HOF-101 and native Cyt c. In this assay, TMB was used as the peroxidase substrate. The enzyme dosages in each group were kept the same (6.67 μg/mL).

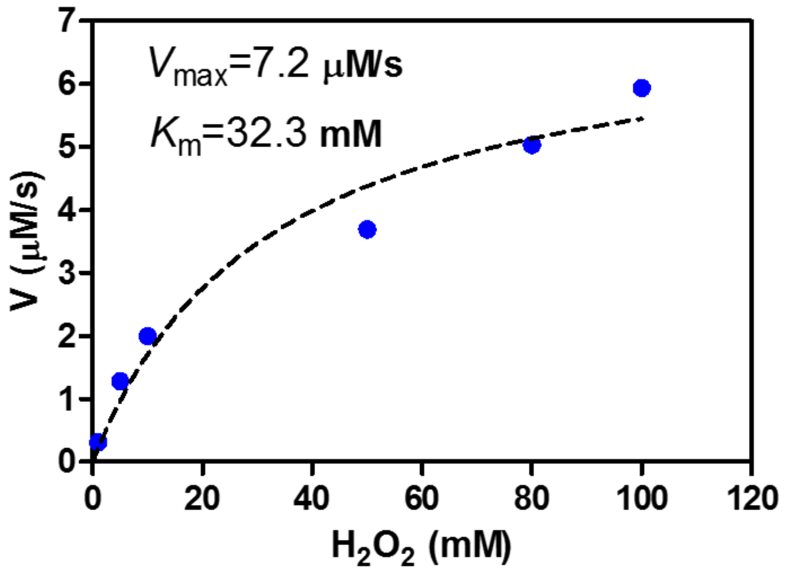

Supplementary Fig. 15. The Michaelis-Menten enzymatic kinetics of CAT-like bioactivity. The dependence of the initial catalytic rates of O_2 generation (V) on the substrate [H_2O_2] concentrations in CAT-like biocatalysis of Cyt c@HOF-101.

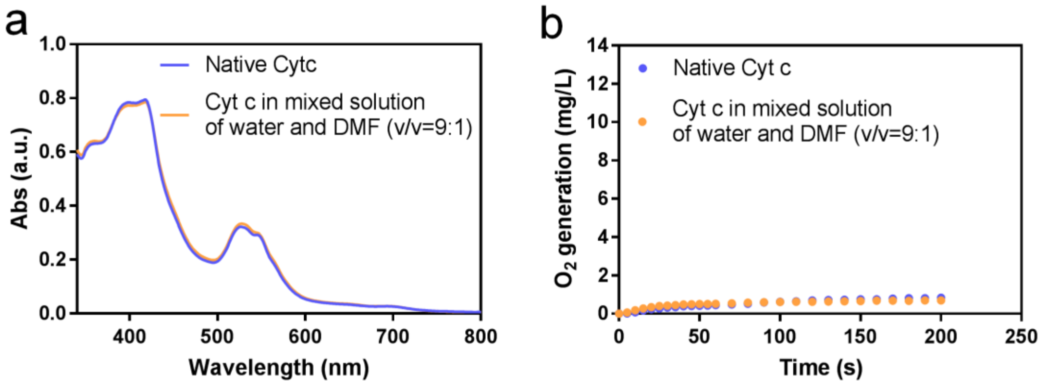

Supplementary Fig. 16. The effect of solution phase on the structure and CAT-like activity of Cyt c. (a) UV-Vis profiles of native Cyt c and the Cyt c after being incubated in the mixed solution of deionized water and DMF (v/v=9:1) for 15 min. The UV-Vis profile of Cyt c was well retained after this treatment, suggesting that the heme structure of Cyt c could not be changed by this exposure. (b) The CAT-like catalytic kinetics of native Cyt c and the Cyt c after being incubated in the mixed solution of deionized water and DMF (v/v=9:1) for 15 min. The result showed that no distinct CAT-like activity was recorded in the resultant Cyt c, further verifying that the solution phase used in the synthetic process could not influence the conformation of Cyt c.

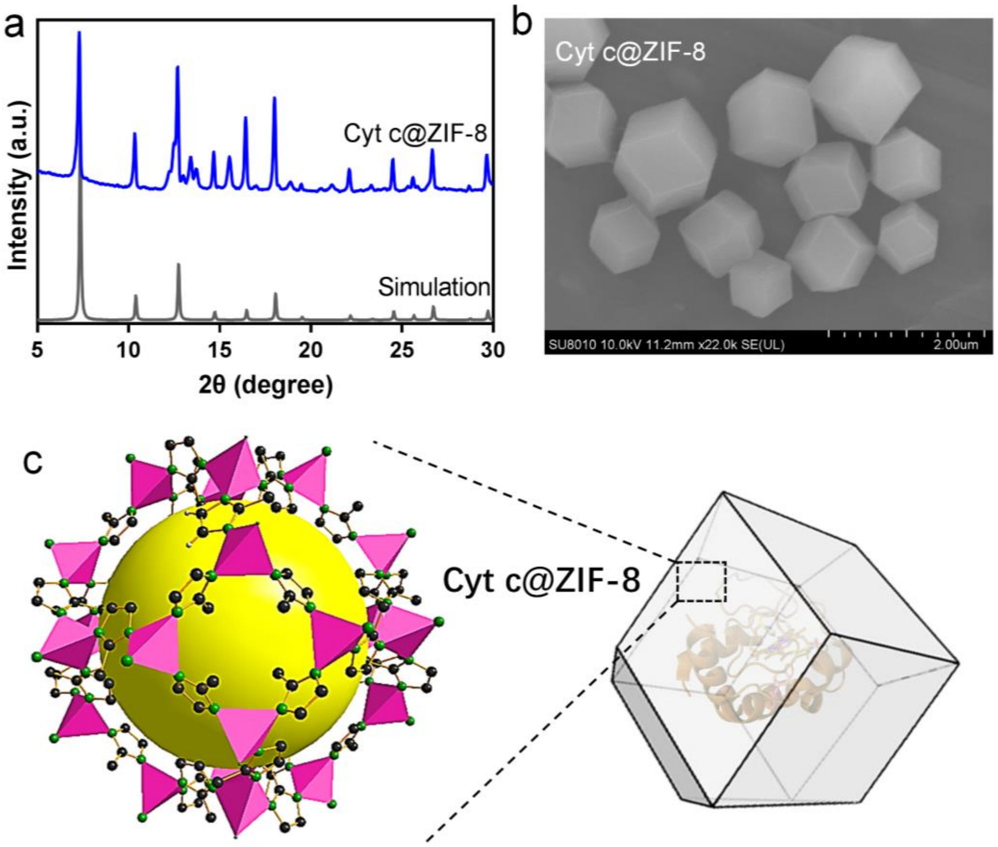

Supplementary Fig. 17. The structure of Cyt c@ZIF-8. (a) The PXRD patterns of Cyt c@ZIF-8 and simulated ZIF-8, respectively. The well maintained PXRD pattern of Cyt c@ZIF-8 suggested that the crystallinity of ZIF-8 was retained after Cyt c encapsulation. (b) The SEM image of Cyt c@ZIF-8. The SEM image further confirmed the high crystallinity of Cyt c@ZIF-8. (c) The structural representation of Cyt c@ZIF-8. The colors used in ZIF-8 are: pink for Zn atom; green for N atom; black for C atom. H atom is removed for clarity.

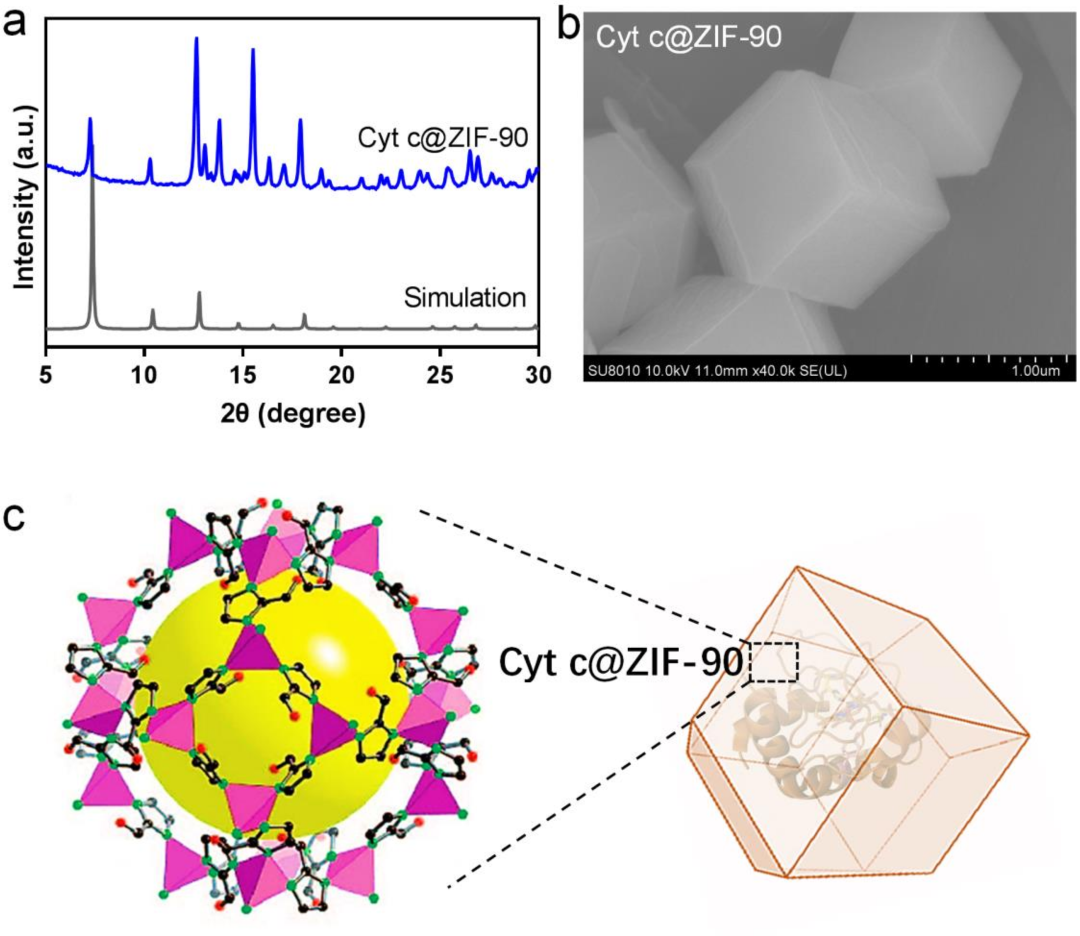

Supplementary Fig. 18. The structure of Cyt c@ZIF-90. (a) The PXRD patterns of Cyt c@ZIF-90, and simulated ZIF-90, respectively. The well maintained PXRD pattern of Cyt c@ZIF-90 suggested that the crystallinity of ZIF-90 was retained after Cyt c encapsulation. (b) The SEM image of Cyt c@ZIF-90. The SEM image further confirmed the high crystallinity of Cyt c@ZIF-90. (c) The structural representation of Cyt c@ZIF-90. The colors used in ZIF-90 are: pink for Zn atom; green for N atom; black for C atom; red for O atom. H atom is removed for clarity.

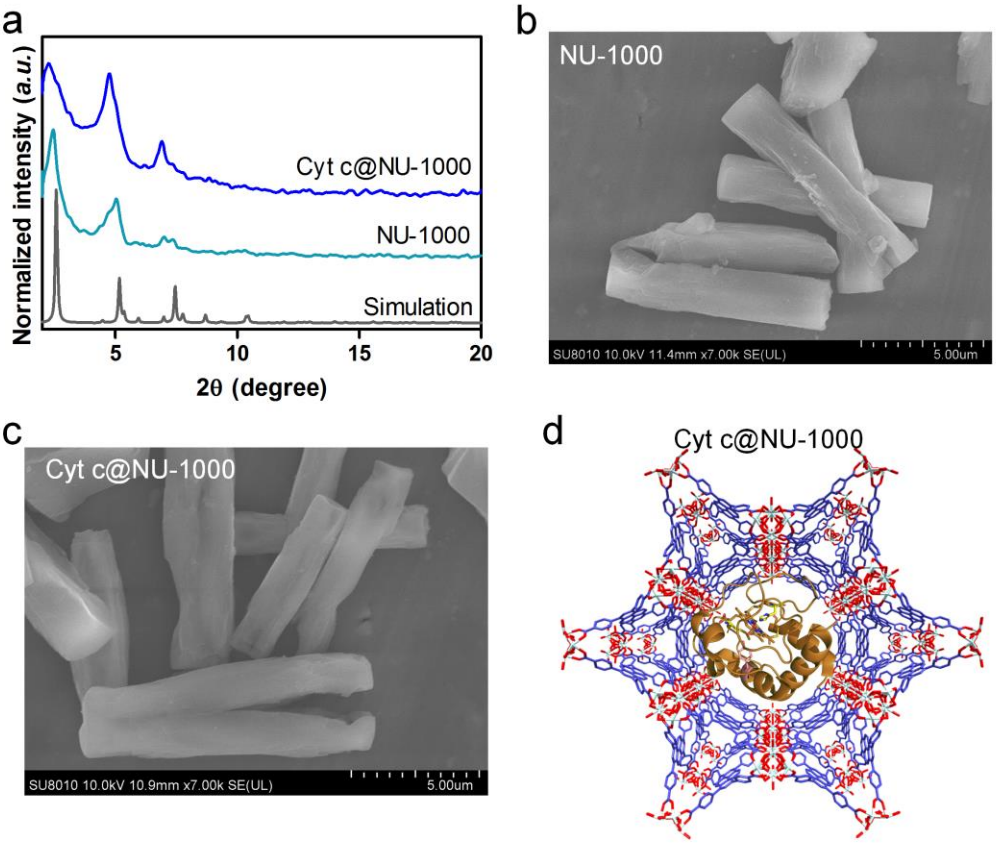

Supplementary Fig. 19. The structure of Cyt c@NU-1000. (a) The PXRD patterns of Cyt c@NU-1000, NU-1000, and simulated NU-1000, respectively. The well maintained PXRD pattern of Cyt c@NU-1000 suggested that the crystallinity of NU-1000 was retained after Cyt c encapsulation. The decreases in relative intensity of the low angle peaks at 2.5° and 5° were caused by the occupancy of large guest molecules (Cyt c) in the mesopores of the NU-1000[2,12]. The SEM images of NU-1000 (b) and Cyt c@NU-1000 (c), respectively. The SEM images further confirmed that the structure of NU-1000 was retained after Cyt c encapsulation. (d) The schematic representation of Cyt c@NU-1000. The colors used in NU-1000 are: red for O atom; blue for C atom; light green for Zr atom; H atom is removed for clarity.

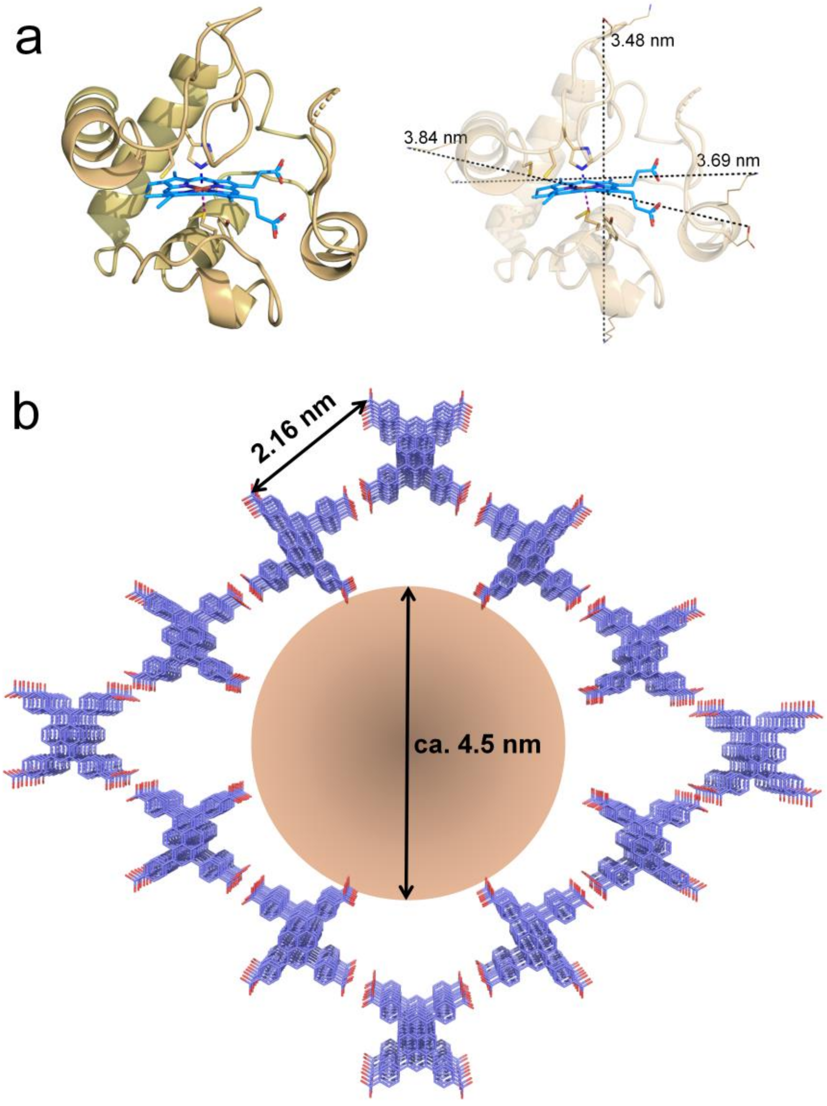

Supplementary Fig. 20. The simulation setup for Cyt c@HOF-101 nanosystem. (a) The structure and molecular size of native Cyt c (PDB: 6k9i). The colors used in heme: light blue for C atom; deep blue for N atom; red for O atom; the central Fe ion is highlighted in orange. (b) The 8-layer tetramer of defective HOFs-101, which was set up for the accommodation of Cyt c. The colors used in HOF-101 are: red for O atom; blue for C atom; H atoms are removed for clarity.

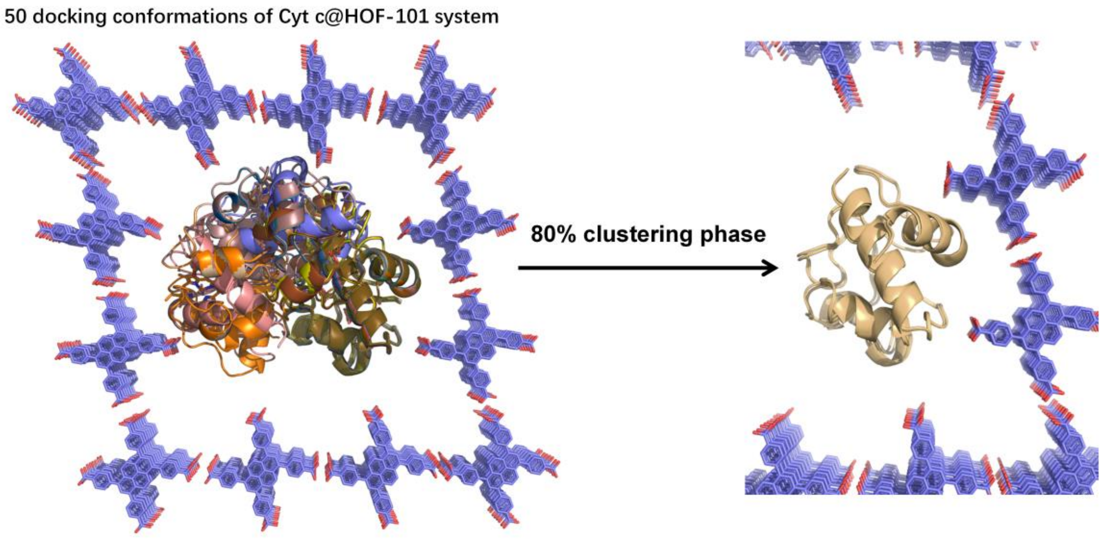

Supplementary Fig. 21. The docking conformations of Cyt c@HOF-101 nanosystem. The potential conformations of Cyt c@HOF-101 and the most clustering phase (80%). The colors used in HOF-101: red for O atom; blue for C atom; H atoms are removed for clarity. A total of 50 conformations of Cyt c@HOF-101 were obtained by the docking process. The docking results showed that Cyt c was extremely concentrated in the defective HOF-101, and the most clustering phase was as high as 80%, with the energy concentrated in the range of ca. 160.0 kJ/mol to ca. 157.0 kJ/mol.

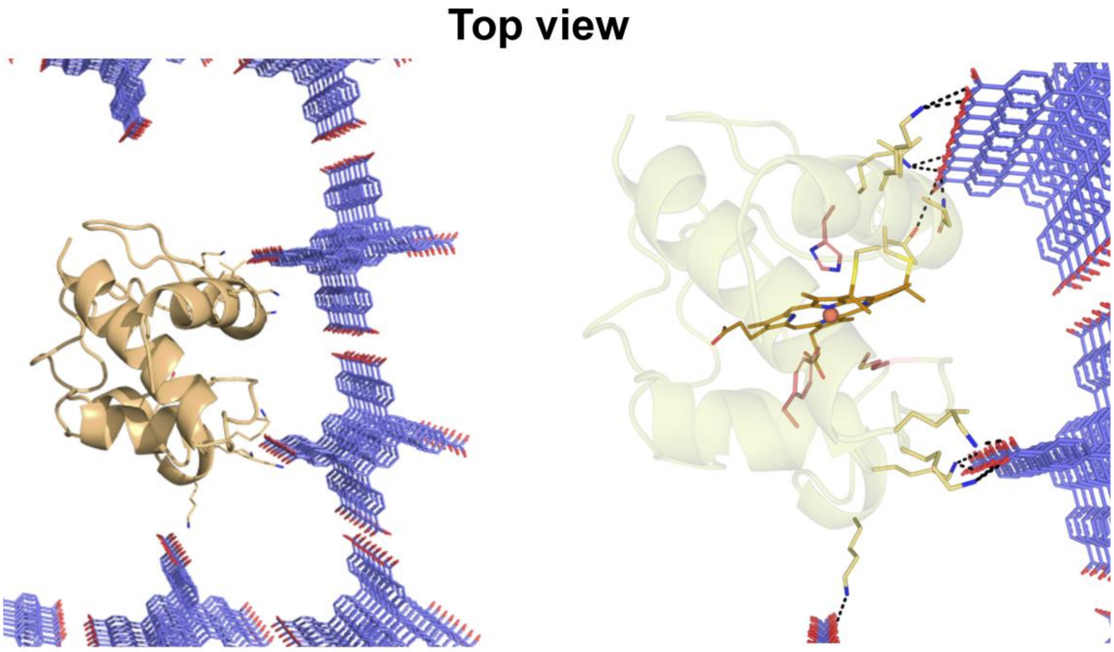

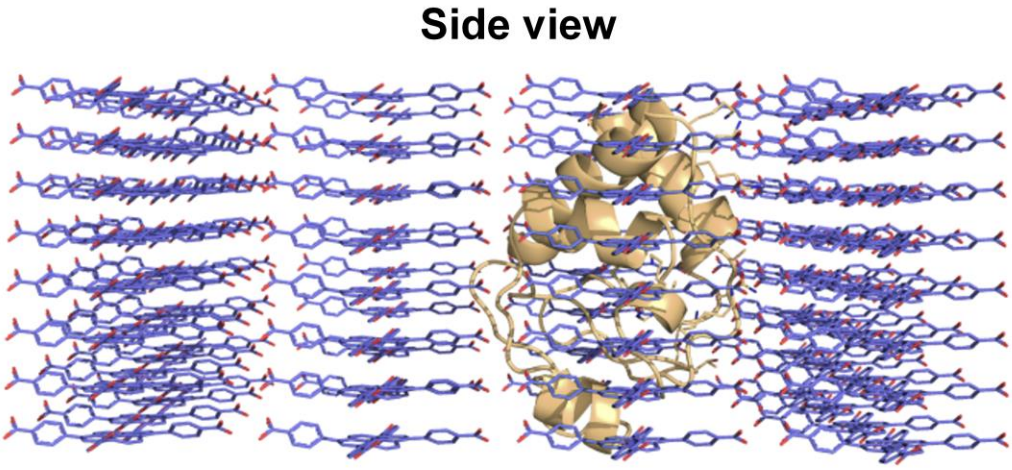

Supplementary Fig. 22. The simulated structure of Cyt c@HOF-101 syste. The top and side views of the structure of Cyt c@HOF-101 based on MD simulation. The colors used in HOF-101 are: red for O atom; blue for C atom; H atoms are removed for clarity. The colors used in Cyt c heme are: brown for C atom; blue for N atom; red for O atom; the central Fe ion is highlighted as an orange ball.

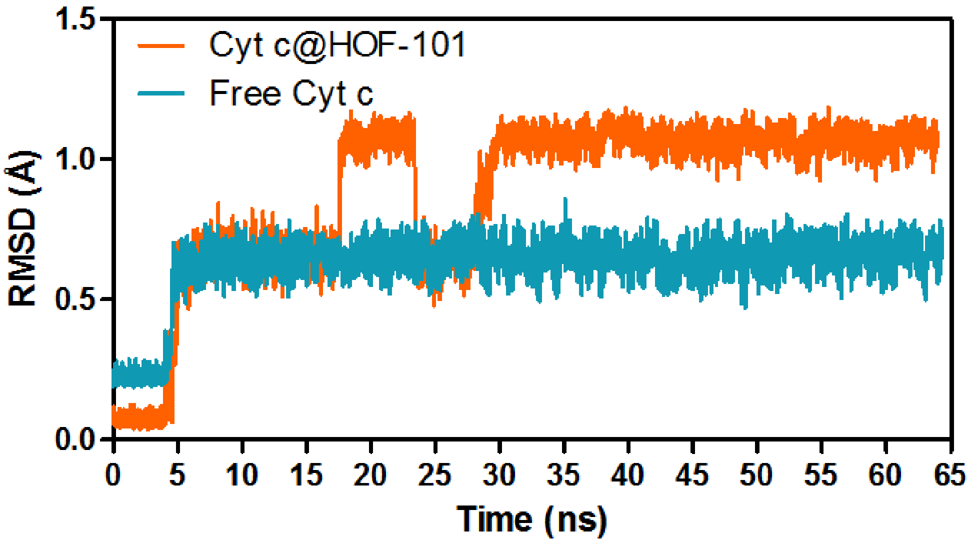

Supplementary Fig. 23. The change of Cyt c backbone after encapsulation. The time-dependent root-mean-square deviation (RMSD) of the Cyt c backbone of free Cyt c and Cyt c@HOF-101 during the MD simulation. For free Cyt c, its RMSD value was unusually stable and almost unchanged in the time range of 5-62 ns. For Cyt c@HOF-101, the RMSD value of Cyt c backbone changed greatly in the time range of 5-30 ns. This suggested that the overall structure of Cyt c was disrupted in this time interval because of the strong interaction between Cyt c and HOF-101. However, the RMSD of Cyt c in Cyt c@HOF-101 became stable after 30 ns, indicating that the overall structure reached a relative equilibrium state.

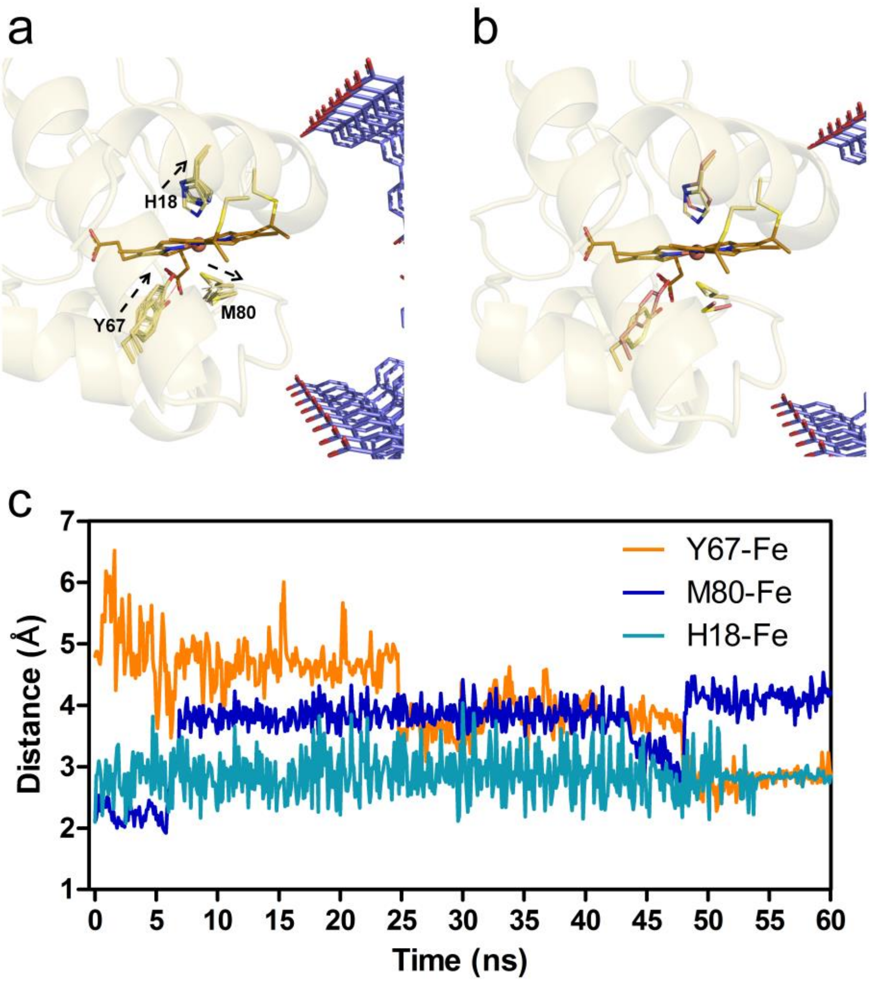

Supplementary Fig. 24. The time-dependent conformation change of heme center in Cyt c@HOF-101 system during the MD simulation. (a) Recording the dynamic trajectories of three important amino acids (H18, T67 and M80) in each 2 ns interval when Cyt c was encapsulated into HOF-101. (b) Comparison of the heme conformations of native Cyt c and the HOF-101-encapsulated Cyt c at a steady state. The colors used in HOF-101 are: red for O atom; blue for C atom; H atoms are removed for clarity. The colors used in Cyt c heme are: brown for C atom; blue for N atom; red for O atom; the central Fe ion is highlighted as an orange ball. (c) The distance changes between the amino acids (H18, T67 and M80) and Fe active site in Cyt c@HOF-101 system during the MD simulation.

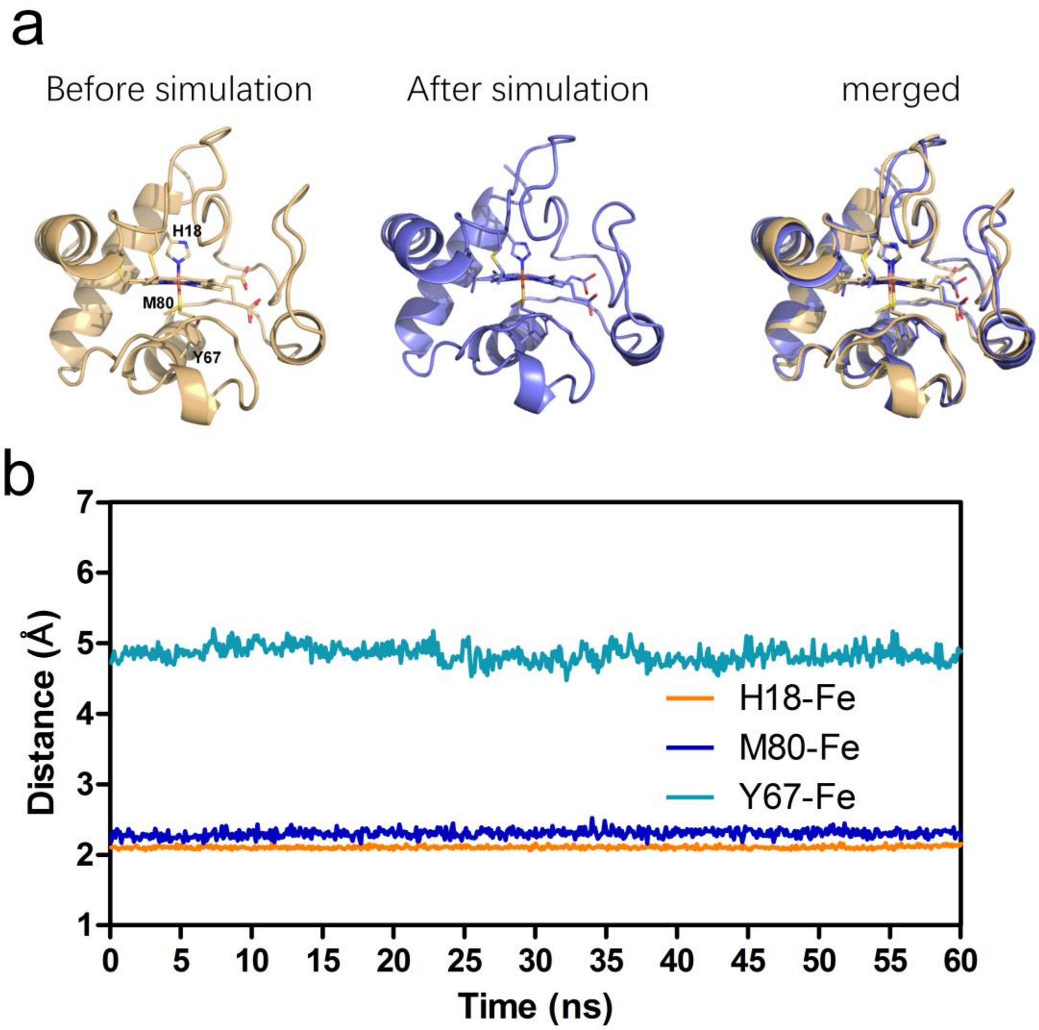

Supplementary Fig. 25. The time-dependent conformation change of heme center in free Cyt c during the MD simulation. (a) The overall structure change of free Cyt c under the similar simulation conditions. (b) The distance changes between the amino acids (H18, T67 and M80) and Fe active site in free Cyt c during the MD simulation. It was observed that the heme conformation could not be changed without the interaction by the designed HOF.

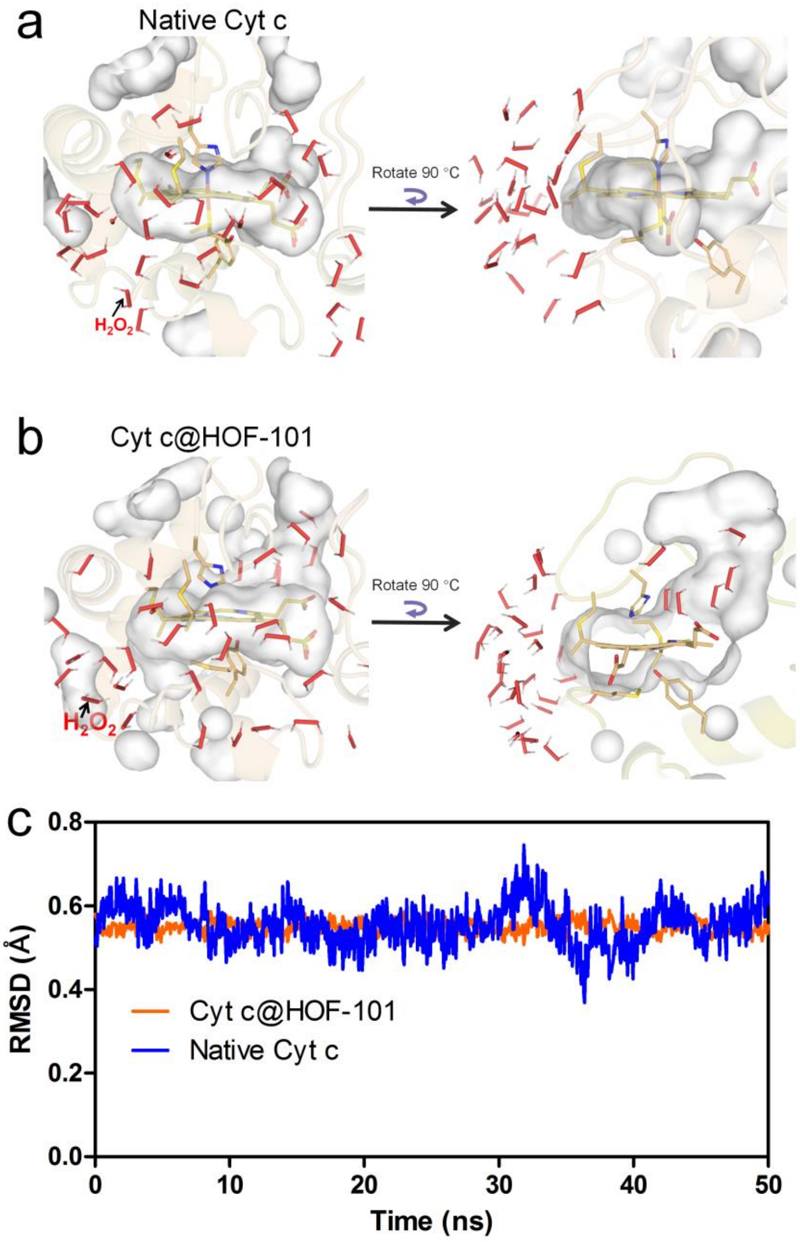

Supplementary Fig. 26. The accessibility of the heme catalytic site. The instantaneous snapshots showed the distribution of H_2O_2 around the catalytic site in free Cyt c (a) and Cyt c@HOF-101 system (b). The H_2O_2 was hard to access the heme in the native conformation of Cyt c because of the narrow and closed binding cavity. However, in Cyt c@HOF-101, the encapsulated Cyt c opened a wider pocket through the interfacial interaction, leading to the high accessibility of the heme region. (c) RMSD of the Cyt c backbones during the simulated process of the H_2O_2 entrance in free Cyt c and Cyt c@HOF-101 system. The RMSD values of Cyt c in the both systems were relatively stable, suggesting that the conformation of Cyt c almost unchanged during the simulated process of the H_2O_2 entrance.

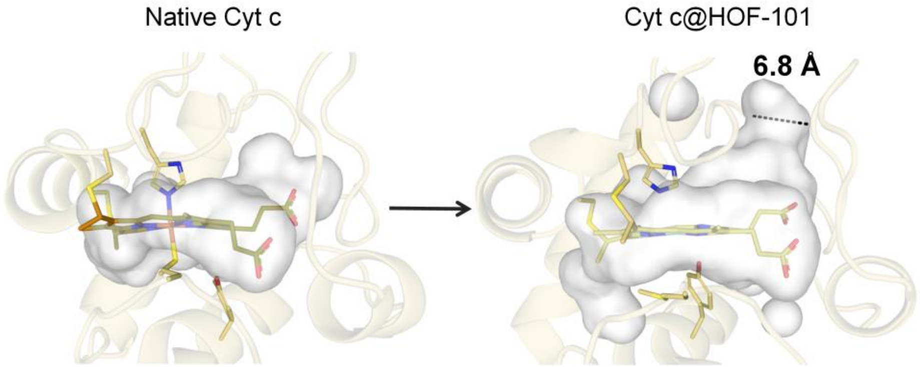

Supplementary Fig. 27. The simulated structure of the heme pocket. The structural features of the heme pocket of Cyt c before and after encapsulation. Under the interfacial interaction by HOF-101, a larger opening pocket (ca. 6.8 Å) was formed in Cyt c@HOF-101, which facilitated the entrance of H_2O_2.

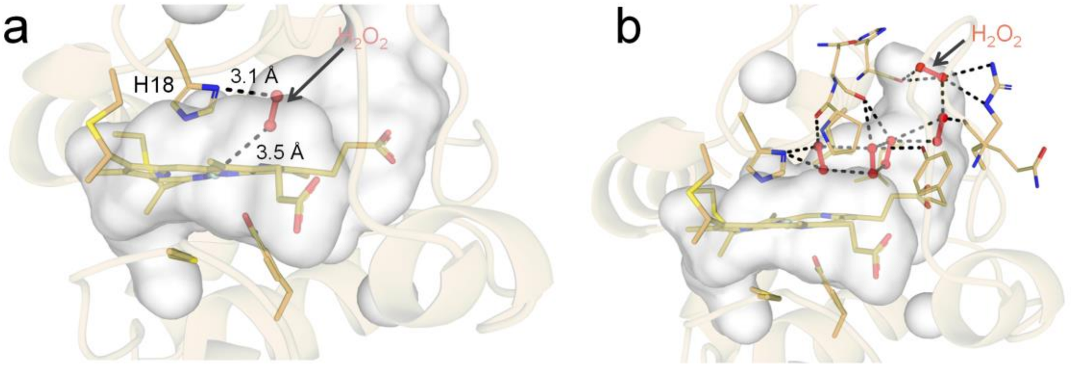

Supplementary Fig. 28. The simulated binding process, and the formed H-bonded network of H_2O_2 in Cyt c@HOF-101. (a) The instantaneous snapshot showed the binding of H_2O_2 with heme, and this H_2O_2 could be stabilized by the distal histidine (H18). (b) The instantaneous snapshot of the formed H-bonded network of H_2O_2, which was stabilized by the polar residues around the changed pocket. Such H-bonded network might also facilitate the proton transfer pathway for CAT catalysis[13].

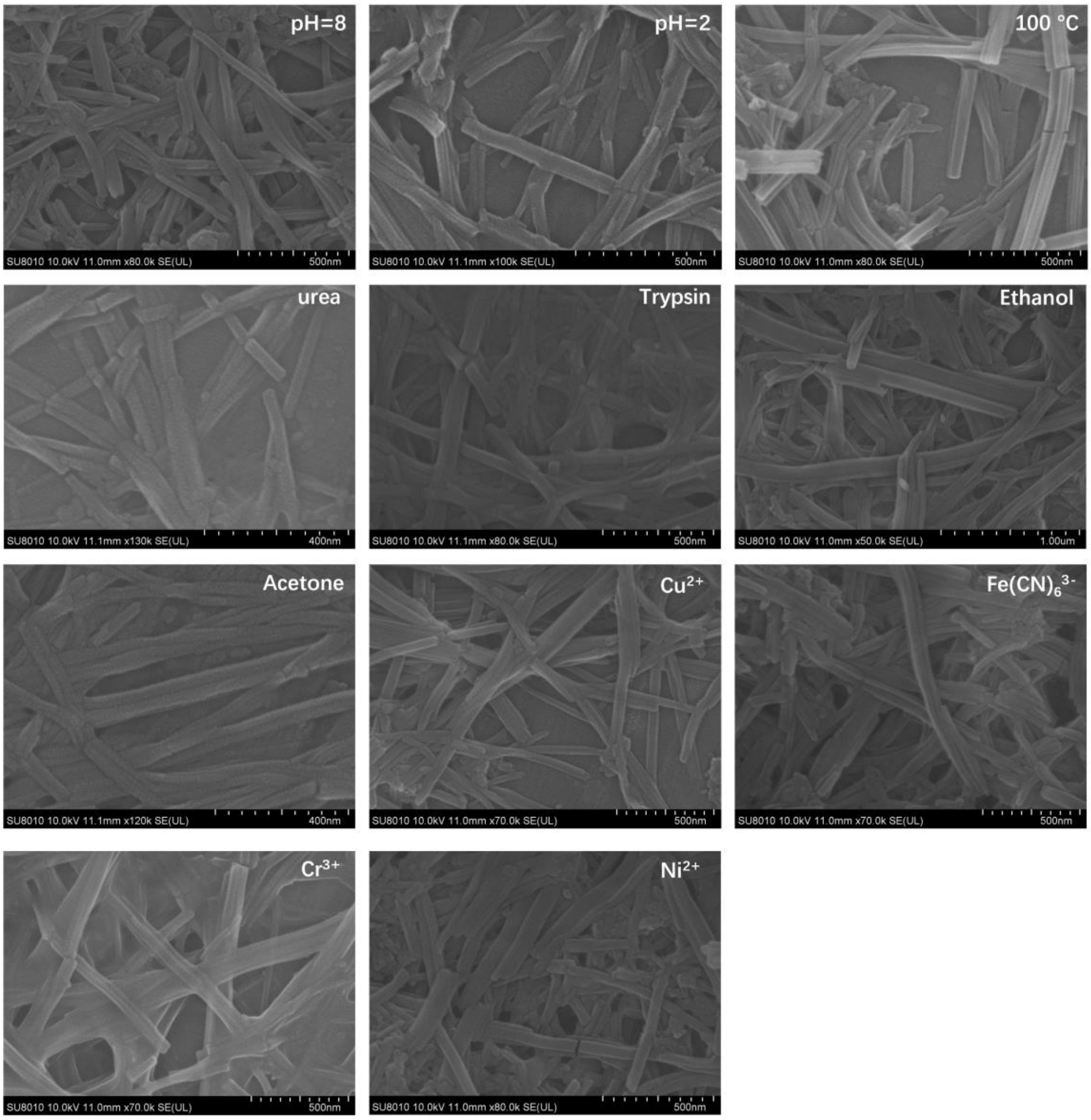

Supplementary Fig. 29. The structural stability of Cyt c@HOF-101. The SEM images of Cyt c@HOF-101 after different conditions treatments.

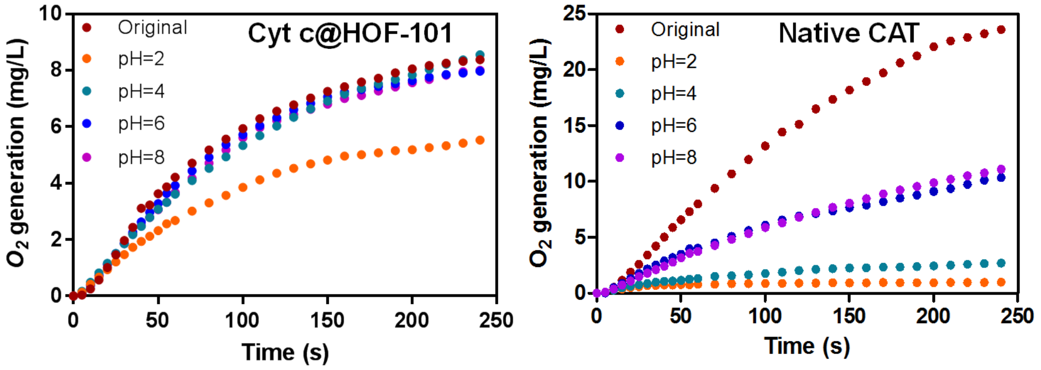

Supplementary Fig. 30. The stability against non-native pH environment. The catalytic kinetics of Cyt c@HOF-101 and native CAT after exposing to non-physiological pH solutions for 30 min. The measurement of bioactivity conversion in Figure 6 (in main text) was based on the change of initial catalytic rate, which was evaluated by the slope of the kinetic curve in the initial phase from 0 to 40 s.

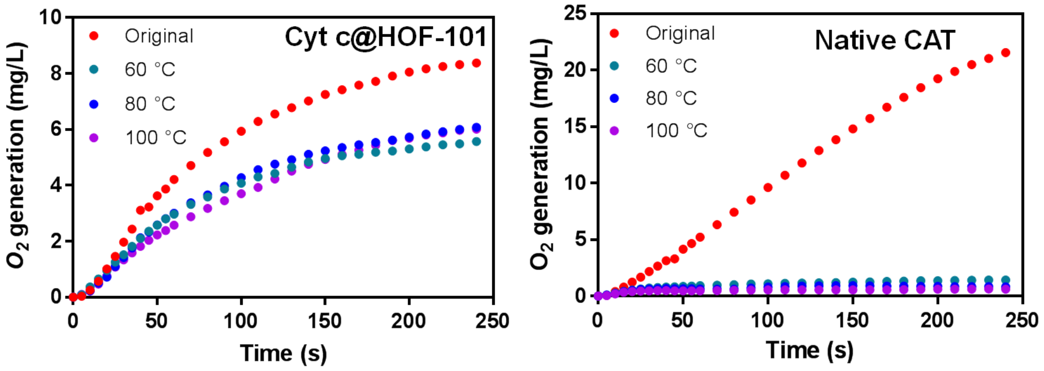

Supplementary Fig. 31. The stability against heating exposure. The catalytic kinetics of Cyt c@HOF-101 and native CAT after heating treatments for 30 min. The measurement of bioactivity conversion in Figure 6 (in main text) was based on the change of initial catalytic rate, which was evaluated by the slope of the kinetic curve in the initial phase from 0 to 40 s.

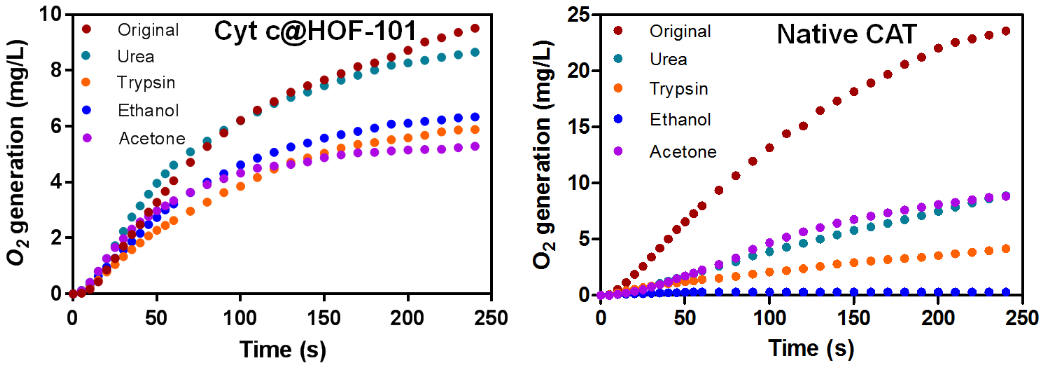

Supplementary Fig. 32. The stability against denaturing reagents and organic solvents. The catalytic kinetics of Cyt c@HOF-101 and native CAT after exposing to different denaturing reagents (urea, hydrolase) and organic solvents for 30 min. The measurement of bioactivity conversion in Figure 6 (in main text) was based on the change of initial catalytic rate, which was evaluated by the slope of the kinetic curve in the initial phase from 0 to 40 s.

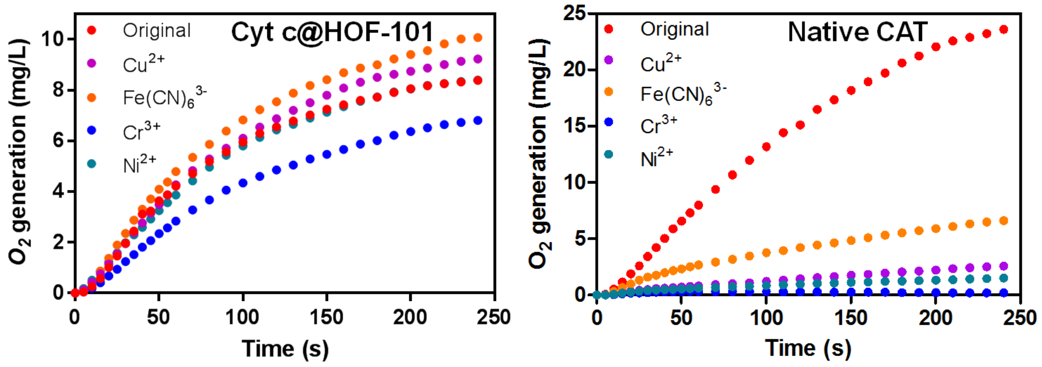

Supplementary Fig. 33. The stability against heavy metal ions. The catalytic kinetics of Cyt c@HOF-101 and native CAT after exposing to different kinds of heavy metal ions for 30 min. The measurement of bioactivity conversion in Figure 6 (in main text) was based on the change of initial catalytic rate, which was evaluated by the slope of the kinetic curve in the initial phase from 0 to 40 s.

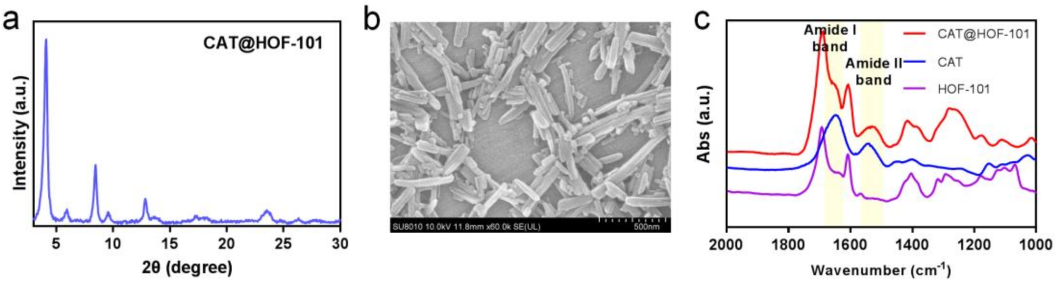

Supplementary Fig. 34. The structural characterizations of CAT@HOF-101. The PXRD (a), SEM image (b) and FT-IR (c) of as-synthesized CAT@HOF-101. The standard Bradford assay gave a ca. 44 wt% CAT loading in CAT@HOF-101.

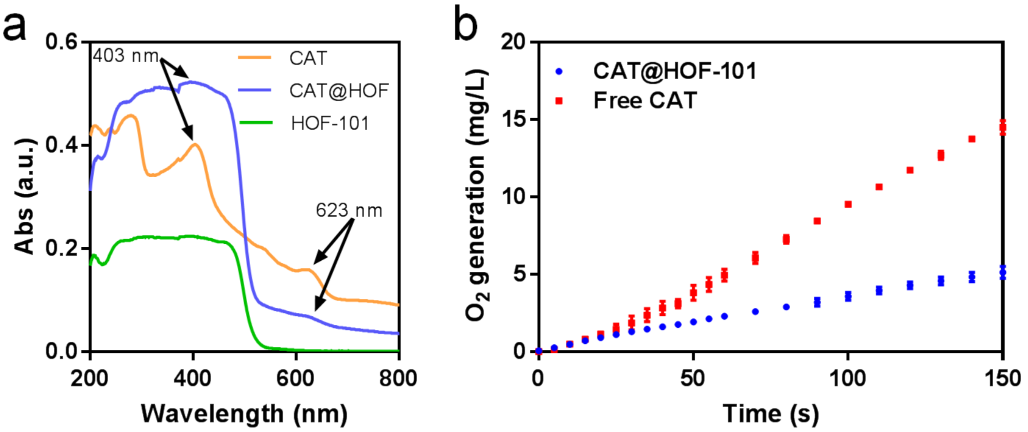

Supplementary Fig. 35. **The UV-Vis DRS and activity of CAT@HOF-101.** (a) The UV-Vis DRS. It revealed that the typical adsorption bands of the heme of CAT were well maintained after the encapsulation. (b) The bioactivity of free CAT and CAT@HOF-101. The CAT dosage in each group was kept at 1 μg/mL. Error bars (SD) are presented, SD = Standard Deviation (n=3). Data are presented as mean values +/- SD.

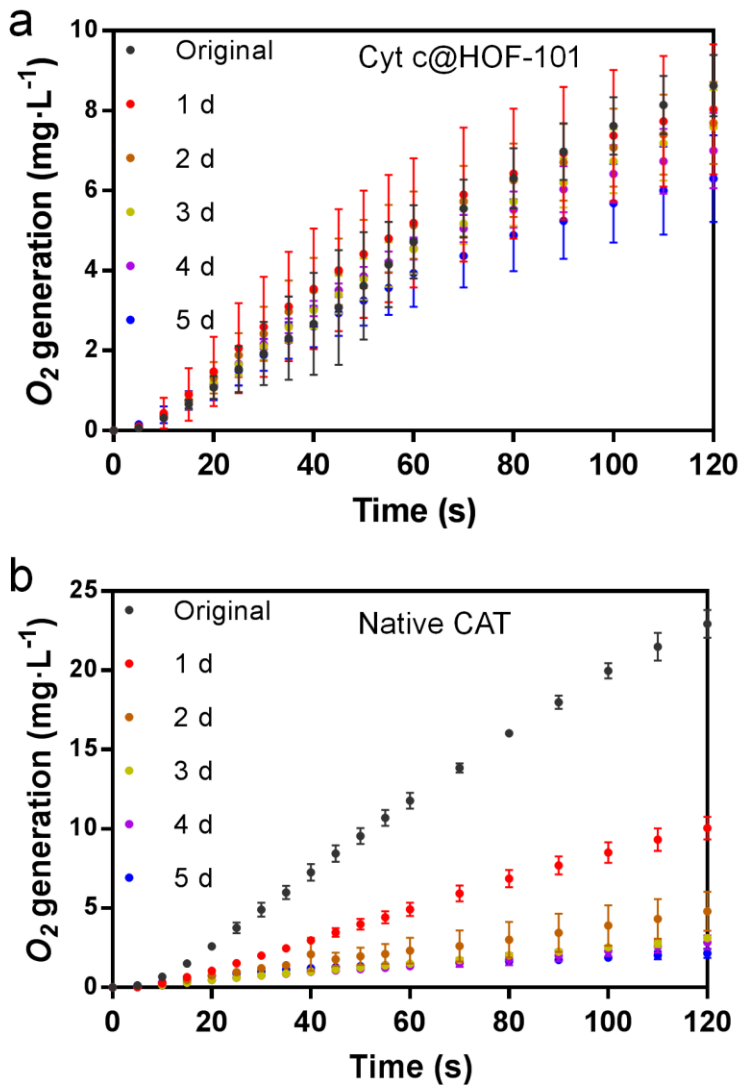

Supplementary Fig. 36. The storage stability. The catalytic kinetics of Cyt c@HOF-101 (a) and native CAT (b) after storage at 35 °C for different periods. The measurement of bioactivity conversion in Figure 6 (in main text) was based on the change of initial catalytic rate, which was evaluated by the slope of the kinetic curve in the initial phase from 0 to 40 s. Error bars (SD) are presented in (a) and (b), SD = Standard Deviation (n=3). Data are presented as mean values +/- SD.

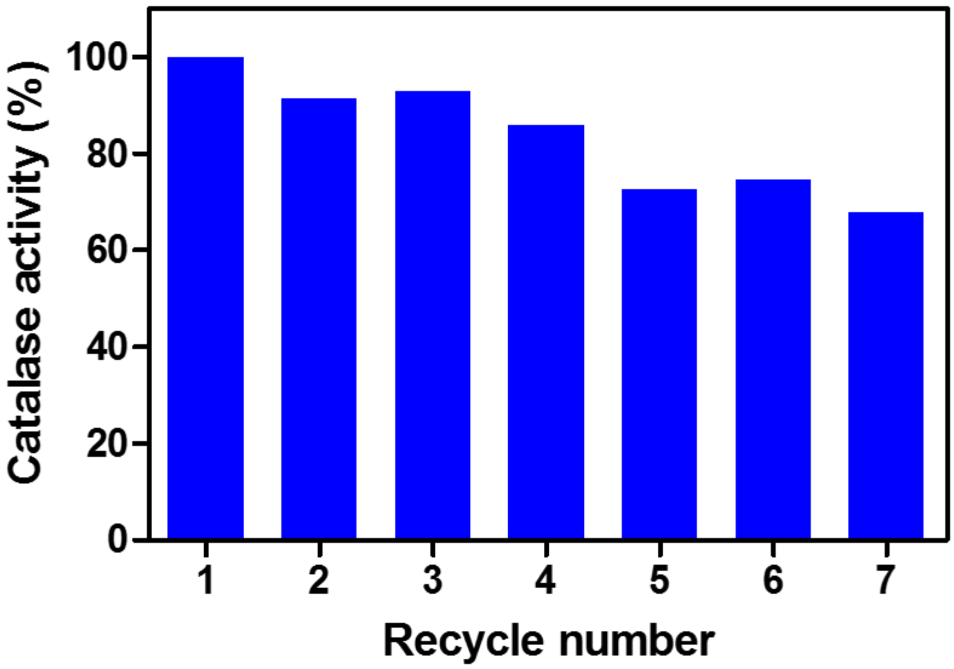

Supplementary Fig. 37. The recyclability. The CAT-like activities of Cyt c@HOF-101 after cycling it for different times.

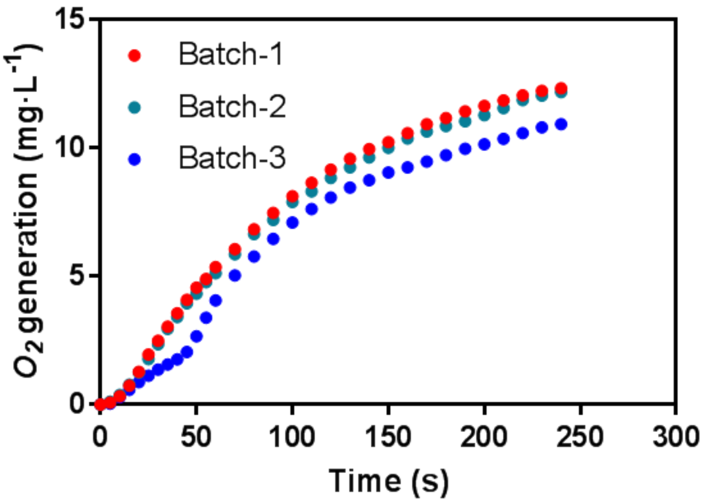

Supplementary Fig. 38. The repeatability of CAT-like activity. The CAT-like bioactivities of Cyt c@HOF-101 synthesized in different batches.

Supplementary References

1. Yin, Q., Zhao, P., Sa, R.-J., Chen, G.-C., Lu, J., Liu, T.-F. & Cao, R. An Ultra-Robust and Crystalline Redeemable Hydrogen-Bonded Organic Framework for Synergistic Chemo-Photodynamic Therapy. *Angew. Chem. Int. Ed.* 57, 7691-7696 (2018).
2. Chen, Y. et al. Insights into the Enhanced Catalytic Activity of Cytochrome c When Encapsulated in a Metal−Organic Framework. *J. Am. Chem. Soc.* 142, 18576−18582 (2020).
3. Sha, F., Chen, Y., Drout, R. J., Idrees, K. B. Zhang, Xuan. & Farha, O. K. Stabilization of an enzyme cytochrome c in a metal-organic framework against denaturing organic solvents. *iScience* 24, 102641 (2021).
4. Islamoglu, T., Otake, K.-i., Li, P., Buru, C. T., Peters, A. W., Akpinar, I., Garibay, S. J. & Farha, O. K. Revisiting the structural homogeneity of NU-1000, a Zr-based metal−organic framework. *CrystEngComm* 20, 5913−5918 (2018).
5. Chen, G., Huang, S., Kou, X., Zhu, F. & Ouyang, G. Embedding Functional Biomacromolecules within Peptide-Directed Metal-Organic Framework (MOF) Nanoarchitectures Enables Activity Enhancement. *Angew. Chem. Int. Ed.* 59, 13947-13954 (2020).
6. Liao, F.-S., Lo, W.-S., Hsu, Y.-S., Wu, C.-C., Wang, S.-C., Shieh, F.-K., Morabito, J.-V., Chou, L.-Y., Wu, K. C.-W. & Tsung, C.-K. Shielding against Unfolding by Embedding Enzymes in Metal−Organic Frameworks via a de Novo Approach. *J. Am. Chem. Soc.* 139, 6530-6533 (2017).
7. Bradford, M. M. A rapid and sensitive method for the quantitation of microgram quantities of protein utilizing the principle of protein-dye binding. *Anal. Biochem.* 72, 248-254 (1976).
8. Fita, T. & Rossmann, M. G. The NADPH binding site on beef liver catalase. *Proc. Natl. Acad. Sci. U. S. A.* 82, 1604−1608 (1985).
9. Feng, D. et al. Stable metal-organic frameworks containing single-molecule traps for enzyme encapsulation. *Nat. Commun.* 6, 5979 (2015).
10. Chen G. et al. Protein-directed, hydrogen-bonded biohybrid framework. *Chem,* 7, 2722-2742 (2021).

11. Secundo, F. Conformational changes of enzymes upon immobilization. *Chem. Soc. Rev.* 42, 6250–6261 (2013).

12. Chen, Y., Li, P., Noh, H., Kung, C. W., Buru, C. T., Wang, X., Zhang, X. & Farha, O. K. Stabilization of Formate Dehydrogenase in a Metal–Organic Framework for Bioelectrocatalytic Reduction of CO_2. *Angew. Chem. Int. Ed.* 58, 7682-7686 (2019).

13. Alfonso-Prieto, M., Biarnés, X., Vidossich, P. & Rovira, C. The Molecular Mechanism of the Catalase Reaction. *J. Am. Chem. Soc.* 131, 11751–11761 (2009).
