# Supplementary material for: Hydrogen-bonded organic framework biomimetic entrapment allowing non-native biocatalytic activity in enzyme
Source: Nat Commun. 2022 Aug 16;13:4816. doi: 10.1038/s41467-022-32454-2 (PMC9381776; doi:10.1038/s41467-022-32454-2)
Supplement: Supplementary file 2 — Reporting Summary [file 41467_2022_32454_MOESM2_ESM.pdf]

nature portfolio

Corresponding author(s): Gangfeng Ouyang

Last updated by author(s): Jun 23, 2022

Reporting Summary

Nature Portfolio wishes to improve the reproducibility of the work that we publish. This form provides structure for consistency and transparency in reporting. For further information on Nature Portfolio policies, see our Editorial Policies and the Editorial Policy Checklist.

Statistics

For all statistical analyses, confirm that the following items are present in the figure legend, table legend, main text, or Methods section.

n/a	Confirmed	
☐	☒	The exact sample size (*n*) for each experimental group/condition, given as a discrete number and unit of measurement
☐	☒	A statement on whether measurements were taken from distinct samples or whether the same sample was measured repeatedly
☒	☐	The statistical test(s) used AND whether they are one- or two-sided<br>*Only common tests should be described solely by name; describe more complex techniques in the Methods section.*
☐	☒	A description of all covariates tested
☐	☒	A description of any assumptions or corrections, such as tests of normality and adjustment for multiple comparisons
☐	☒	A full description of the statistical parameters including central tendency (e.g. means) or other basic estimates (e.g. regression coefficient) AND variation (e.g. standard deviation) or associated estimates of uncertainty (e.g. confidence intervals)
☒	☐	For null hypothesis testing, the test statistic (e.g. *F*, *t*, *r*) with confidence intervals, effect sizes, degrees of freedom and *P* value noted<br>*Give P values as exact values whenever suitable.*
☒	☐	For Bayesian analysis, information on the choice of priors and Markov chain Monte Carlo settings
☒	☐	For hierarchical and complex designs, identification of the appropriate level for tests and full reporting of outcomes
☒	☐	Estimates of effect sizes (e.g. Cohen's *d*, Pearson's *r*), indicating how they were calculated

Our web collection on statistics for biologists contains articles on many of the points above.

Software and code

Policy information about availability of computer code

Data collection	No computer code was available. All-atom explicit solvent molecular dynamics simulation was carried out by NAMD software (version 2.15); cryo-EM images were collected by Thermofisher Scientific Titan Krios G3i electron microscope (FEI Titan Krios G3i (D3845)),and data collected was performed using Serial EM (version 3.8). Fluorescence images were collected by confocal laser scanning microscope (LSM 880 NLO, Carl Zeiss, Göttingen, Germany). The SEM morphology images of were analyzed by a ultra-high revolution field emission scanning electron microscope (SU8010, Hitachi, Japan). Other routine material characterizations were stated in the Supplementary Information.
Data analysis	GraphPad Prism 5 (version 5.0.1), Microsoft Excel (version 2016) and DigitalMicrograph (Gatan) software (version 3.23.1518.0) were used for data analysis

For manuscripts utilizing custom algorithms or software that are central to the research but not yet described in published literature, software must be made available to editors and reviewers. We strongly encourage code deposition in a community repository (e.g. GitHub). See the Nature Portfolio guidelines for submitting code & software for further information.

Data

Policy information about availability of data

All manuscripts must include a data availability statement. This statement should provide the following information, where applicable:

- Accession codes, unique identifiers, or web links for publicly available datasets
- A description of any restrictions on data availability
- For clinical datasets or third party data, please ensure that the statement adheres to our policy

All data supporting this study and its findings are available within the article and its Supplementary Information or from the corresponding authors upon request. The Cyt c structure used herein is available in the PDB database under accession code 6k9i [https://www.rcsb.org/structure/6K9I]. The structure of GroEL-GroES

chaperonin used herein is available in the PDB database under accession code 1pf9 [https://www.rcsb.org/structure/1pf9]. The CAT structure used herein is available in the PDB database under accession code 7di8 [https://www.rcsb.org/structure/7di8].

Field-specific reporting

Please select the one below that is the best fit for your research. If you are not sure, read the appropriate sections before making your selection.

- [x] Life sciences
- [ ] Behavioural & social sciences
- [ ] Ecological, evolutionary & environmental sciences

For a reference copy of the document with all sections, see nature.com/documents/nr-reporting-summary-flat.pdf

Life sciences study design

All studies must disclose on these points even when the disclosure is negative.

Sample size	This study was not involved any cell experiment. All enzymatic activity assays were performed with the sample size, n=3. All three sample in each enzymatic assay showed similar results. All sample sizes and error bars were listed in the corresponding figure legends or on the figures.
Data exclusions	No data were excluded from the analyses.
Replication	The enzymatic activity assays were performed three times in the case of the same experimental materials, experimental conditions and experimental. All replications were successful. In addition, the number of repeats were provided in each figure caption and experimental sections. For electronic microscopy data in the main text (Figs. 1b-c, e) and Supplementary Information (Supplementary Figs. 8a-b; 9a-d; 10; 17b; 18b; 19b-c; 29; 34b), each experiment was repeated more than three times. All replications were successful. This has been stated in the Data availability statement.
Randomization	The allocation of the samples was random.
Blinding	Blinding was not neccesary for this study, because this work focused on the Cyt c activities mediated by HOF-101, and did not investigate more than two populations.

Reporting for specific materials, systems and methods

We require information from authors about some types of materials, experimental systems and methods used in many studies. Here, indicate whether each material, system or method listed is relevant to your study. If you are not sure if a list item applies to your research, read the appropriate section before selecting a response.

Materials & experimental systems

n/a	Involved in the study	
x		Antibodies
x		Eukaryotic cell lines
x		Palaeontology and archaeology
x		Animals and other organisms
x		Human research participants
x		Clinical data
x		Dual use research of concern

Methods

n/a	Involved in the study	
x		ChIP-seq
x		Flow cytometry
x		MRI-based neuroimaging
